# Effects of a Flaxseed-Derived Lignan Supplement in Type 2 Diabetic Patients: A Randomized, Double-Blind, Cross-Over Trial

**DOI:** 10.1371/journal.pone.0001148

**Published:** 2007-11-07

**Authors:** An Pan, Jianqin Sun, Yanqiu Chen, Xingwang Ye, Huaixing Li, Zhijie Yu, Yanfang Wang, Wenjia Gu, Xinyi Zhang, Xiafei Chen, Wendy Demark-Wahnefried, Yong Liu, Xu Lin

**Affiliations:** 1 Key Laboratory of Systems Biology, Institute for Nutritional Sciences, Shanghai Institutes for Biological Sciences, Chinese Academy of Sciences and Graduate School of the Chinese Academy of Sciences, Shanghai, China; 2 Huadong Hospital, Fudan University, Shanghai, China; 3 University of Texas-MD Anderson Cancer Center, Houston, Texas, United States of America; University of Toronto, Canada

## Abstract

**Background:**

Flaxseed consumption has been shown to improve blood lipids in humans and flaxseed-derived lignan has been shown to enhance glycemic control in animals. The study aimed to investigate the effect of a flaxseed-derived lignan supplement on glycemic control, lipid profiles and insulin sensitivity in type 2 diabetic patients.

**Methodology/Principal Findings:**

This was a randomized, double-blind, placebo-controlled, cross-over trial and it was conducted between April and December 2006 in Shanghai, China. Seventy-three type 2 diabetic patients with mild hypercholesterolemia were enrolled into the study. Patients were randomized to supplementation with flaxseed-derived lignan capsules (360 mg lignan per day) or placebo for 12 weeks, separated by an 8-week wash-out period. HbA1c, lipid profiles, insulin resistance index and inflammatory factors were measured. Sixty-eight completed the study and were included in the analyses. The lignan supplement significantly improved glycemic control as measured by HbA_1c_ (-0.10±0.65 % vs. 0.09±0.52 %, *P* = 0.001) compared to placebo; however, no significant changes were observed in fasting glucose and insulin concentrations, insulin resistance and blood lipid profiles. Urinary excretion of lignan metabolites (enterodiol and enterolactone) was significantly higher after the lignan supplement intervention compared to baseline (14.2±18.1 vs. 1.2±2.4 µg/mL, *P*<0.001). Data also suggested minimal competition between lignan and isoflavones for bioavailability when measured by the excretion concentrations.

**Conclusions/Significance:**

Daily lignan supplementation resulted in modest, yet statistically significant improvements in glycemic control in type 2 diabetic patients without apparently affecting fasting glucose, lipid profiles and insulin sensitivity. Further studies are needed to validate these findings and explore the efficacy of lignans on type 2 diabetes.

**Trial Registration:**

ClinicalTrials.gov NCT00363233

## Introduction

Type 2 diabetes is a serious and economically devastating disease. Over the past few decades its worldwide prevalence has increased dramatically, especially in the developing countries such as China and India [1]. Diabetic patients with poor glycemic control suffer greatly from micro- and macro-vascular complications, especially cardiovascular diseases (CVD), the major cause of mortality in this patient population [2]. Furthermore, high prevalence of dyslipidemia in type 2 diabetic patients also contributes to elevated CVD risk [3].

Recently, phytoestrogens have gained increasing attention because of accumulating evidence suggesting their protective roles against numerous chronic diseases, including cancers, CVD, dyslipidemia and diabetes [4], [5]. To date, the most well-studied phytoestrogens are isoflavones and lignans. Dietary lignans are broadly available in plant-based foods, particularly concentrated in flaxseed [4]. Several randomized controlled studies showed beneficial effects of a flaxseed-supplemented diet (30–50 g/day) on lipid profiles in both normal- and hypercholesterolemic subjects [6]–[10]. Recently, two studies reported that secoisolariciresinol diglucoside (SDG), the major plant lignan in flaxseed [11], significantly reduced total cholesterol and LDL cholesterol (LDL-C) concentrations in rabbits [12], [13]. However, null findings were observed in a recent study of a flaxseed-derived lignan supplement (500 mg/day) on lipid profiles in 22 healthy women [14]. Evidence of flaxseed or lignans on glucose metabolism is limited. One pilot study reported that flaxseed mucilage significantly decreased postprandial blood glucose in young healthy volunteers [15]. In another clinical trial, flaxseed supplements were also found to decrease glucose and insulin levels in 25 hypercholesterolemic postmenopausal women [16]. Data related to lignan supplements on diabetes has been documented exclusively from animal studies in which SDG significantly prevented or delayed the onset of diabetes and improved glycemic control in rats with type 1 [17] and type 2 diabetes [18].

To our knowledge, no clinical trials thus far have determined the effect of SDG on glycemic control or lipid profiles in type 2 diabetic patients. This is particularly intriguing among Asians who already consume high intakes of soy-derived isoflavone and may respond differently to lignan supplements compared to Westerners [19]. Therefore, we evaluated the effect of a flaxseed-derived lignan supplement (containing 360 mg/day SDG, equivalent to 27–60 g of whole flaxseed [20]) on indexes of glycemic control, insulin resistance and lipid profiles in a randomized, double-blind, placebo-controlled, cross-over study. The dosage was chosen based upon the dose range of flaxseed which has been used safely and effectively in previous reported flaxseed clinical trials on dyslipidemia [6]–[10].

## Methods

The protocol for this trial and supporting CONSORT checklist are available as supporting information; see Protocol S1 and Checklist S1.

### Participants

A total of 581 type 2 diabetic patients were screened for inclusion at the local community medical service centers in urban districts of Shanghai. Participants were considered eligible if they met the following criteria: 1) 50–79 years of age (women were required to be postmenopausal for at least 1-year); 2) LDL-C level ≥2.9 mmol/L; and 3) diagnosis of type 2 diabetes, but not using exogenous insulin for glycemic control. Exclusion criteria were: 1) current or previous (preceding 6 months) estrogen-use; 2) regularly taking phytoestrogen-containing supplements; 3) antibiotic-use in the preceding 3 months; 4) severe renal, liver, heart, pituitary, thyroid or mental diseases, alimentary tract ulceration or diseases affecting absorption; or 5) history of cancer, history of drug or alcohol abuse.

### Ethics

The study protocol was approved by the Ethics Committee of Institute for Nutritional Sciences, Chinese Academy of Sciences. All participants provided signed informed consents.

### Interventions

Thirty-seven subjects began lignan supplements and 36 subjects started on the placebo for 12 weeks. After an 8-week wash-out period, the participants received the alternative treatment for another 12 weeks. While on study, participants were instructed to maintain their habitual diets, levels of physical activity, and use of prescribed medications. During the 12 weeks, participants assigned to the lignan supplements were instructed to take three lignan capsules (0.6 g/capsule) each day that provided a daily amount of 360 mg isolated flaxseed lignan. The raw materials of lignan were donated by Frutarom Netherlands BV (LinumLife™ Extra, Veenendaal, The Netherlands) and were produced by Jarrow Formulas Inc (Flax Essence™ , Los Angeles). The three capsules provided 3.7 kilocalories and were comprised of 20% SDG, 15.6% fat, 3.2% protein, 2.6% fiber and 30% carbohydrate. Participants randomly assigned to the placebo group were instructed to take 3 placebo capsules per day, which were comprised of rice flour (98%) and provided a total of 5.8 kilocalories. Subjects were asked to return any unused capsules and adherence was assessed by pill counts, as well as by urinary concentrations of lignan metabolites (for SDG-specific adherence).

### Objectives

The objective of the study was to investigate the effect of a flaxseed-derived lignan supplement on indexes of glycemic control, insulin resistance and lipid profiles in type 2 diabetic patients.

### Outcomes

All participants were scheduled to visit Huadong Hospital every 3 weeks to obtain new capsules, and their adherence and physical status were evaluated. Dietary intakes were assessed using 3-day food records which ascertained intakes during 2-weekdays and 1-weekend day at four timepoints throughout the study (1 week prior to and during the last week of each intervention period). All food records were reviewed for completeness and coded by the trained dietitians who were blinded to the study arms. Energy and nutrient intakes were calculated using the SY Nutrition Software (Fudan University, Shanghai, China) based on the local food composition database. Physical activity level was evaluated by asking the average times per week spent on several common activities (e.g. running, jogging, dancing, bicycling) in the last month and each activity was assigned a metabolic equivalent value (MET) according to accepted standards [21]. After fasting overnight, blood pressure and anthropometric parameters (height, weight, waist and hip circumference) were measured and blood samples were collected at the beginning and the end of each two intervention periods. Fasting venous blood samples were collected in 7-mL EDTA-treated vacutainers for insulin analysis and 7-mL serum separator vacutainers for measurements of fasting glucose and lipid profiles. After centrifugation at 2600 × g for 10 min at 4°C, samples were aliquotted and stored at −80°C until batch analysis. Fasting morning urine samples (50 mL) were collected at identical timepoints using plastic jugs containing 50 mg ascorbic acid. Urine samples were aliquotted and stored at −20°C until processed.

Serum total cholesterol, HDL cholesterol (HDL-C), LDL-C, triacylglycerol, and glucose were measured using reagents purchased from Wako Pure Chemical Industries (Osaka, Japan), serum lipoprotein(a) [Lp(a)], apolipoprotein A-1 and B (apoA1 and apoB) were measured using kits from Roche Diagnostics (Mannheim, Germany). All the above assays were performed on an automatic analyzer (Hitachi 7080, Japan) within one day. HbA_1c_ was determined by turbidometric immunoinhibition on packed red blood cells on the automatic analyzer using kits from Roche Diagnostics [22]. This assay is approved by the US National Glycohemoglobin Standardization Program and by the Food and Drug Administration for clinical use. Plasma insulin was determined by radioimmunoassay (Linco Research, MO). Insulin resistance was calculated using the Homeostasis Model Assessment of Insulin Resistance method (HOMA-IR) = (Insulin (µU/mL)×glucose (mmol/L))/22.5 [23]. Urinary excretion of lignan metabolites (enterodiol and enterolactone) and isoflavones (genistein and daidzein) was measured using a modified HPLC method [24], [25]. The intra- and inter-assay CVs ranged from 2.3% to 15.8% for enterodiol, enterolactone, genistein and daidzein at concentrations of 1.0 and 10 µg/mL.

Lp(a) concentrations were measured in 245 serum samples due to reagent shortage. The values of urinary excretion of lignan or isoflavone metabolites were replaced by 0.05 µg/mL (half of the lowest detectable limit) when not detectable.

### Sample Size

As there were no extant studies that had investigated the effect of flaxseed-derived lignans on HbA1c, the sample size was calculated based on the previous studies of flaxseed on LDL-C concentrations. Using a one-sided 5% significance level, a sample of 67 patients was needed for this cross-over trial, assuming a 10% drop-out rate. This gave the study 90% power to detect a 5% difference (0.2 mmol/L) in LDL-C between treatments (assuming a common SD of 0.5 mmol/L).

### Randomization and Blinding

The random allocation sequence was developed using computer program by a statistician who was not involved in the study. After enrollment, each participant was randomly given a unique study number. Randomization and allocation to the treatment or placebo group was based on the study number. Participants were randomized to the intervention or the placebo arms using stratification factors of gender and tertiled LDL-C concentrations from screening. In detail, women and men were sorted by their levels of LDL-C, respectively. Then randomization was performed using a block size of 3 and length of 10 for men and length of 16 for women, respectively. Placebo capsules were almost identical to the lignan capsules in size, shape, color, and taste. Each bottle of capsules was marked with the participant's study number, but no product identifier. Participants, health professionals, statisticians, and other research staff involved in the trial were blinded to the assignments.

### Statistical Methods

Differences between values after 12-week intervention were analyzed in Stata 9.2 (Stata ™, Texas) using a mixed model analysis of covariance with treatment (lignan or placebo) and period (first or second period) as fixed factors, subjects as random factors, and baseline measurements as covariates. Further fixed terms corresponding to treatment/period interactions were included to test for any carry-over effect between periods, and treatment/covariate interaction was included to test whether the treatment effect of flaxseed lignan varied according to the baseline values of the covariate. Given the weight-dependent nature of the study endpoints, weight and weight changes were incorporated into the model as covariates. However the results were not affected and the data are therefore not shown. Data that were not normally distributed, as assessed by the Shapiro-Wilks test, were natural-logarithmically transformed prior to analysis (see table footnotes for details). Differences before and after treatments were analyzed using paired Student's t-test. Pre-post differences in physical activity level, and habitual energy and nutrient intake were analyzed using ANOVA. Differences were considered significant at *P*<0.05.

## Results

### Participant Flow, Recruitment and Numbers Analyzed

**Figure 1:**
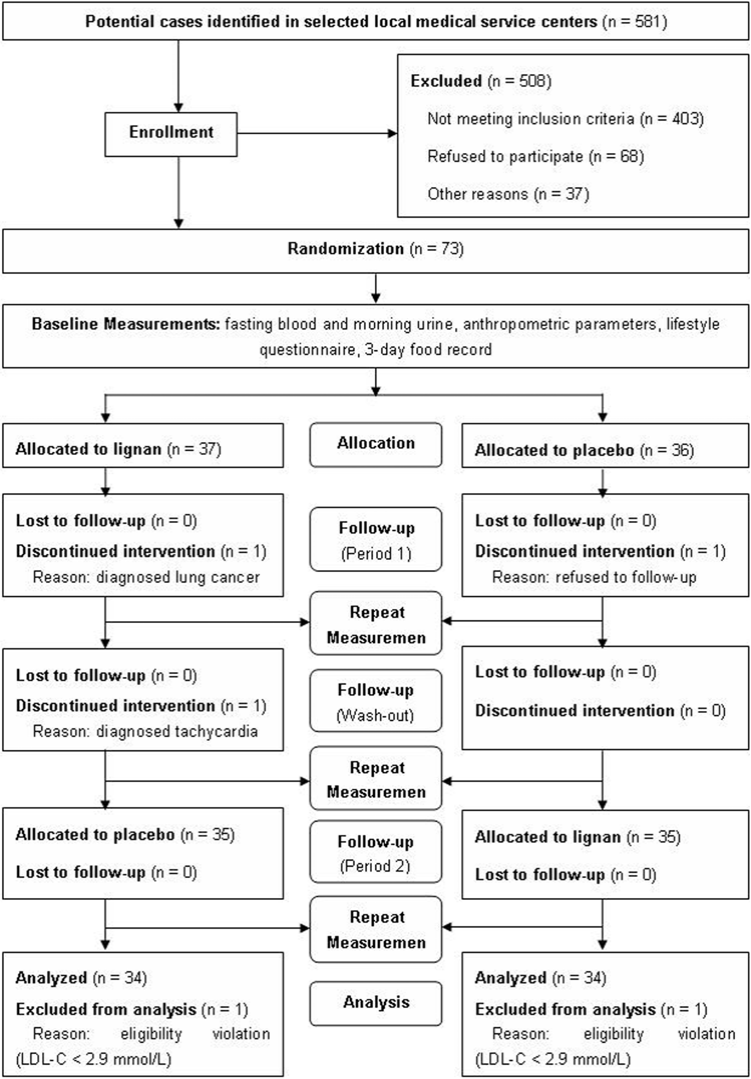
Study flow chart of the lignan intervention trial.

A total of 581 type 2 diabetic patients were screened for inclusion in the study (Figure 1). All participants were enrolled between December 2005 and April 2006 in urban districts of Shanghai. The participants were initially screened via their health archives with the help of the local community medical service centers. Then a screening questionnaire and physical examination were performed to evaluate eligibility. Finally, 73 subjects agreed to participate. Of the 73 subjects, 70 completed the study and 68 were available for the final full analysis set (FAS). One subject receiving lignan capsules was diagnosed with lung cancer one week after the study began and decided to withdraw, one subject receiving placebo withdrew one week after the study began because of poor physical status, and one subject withdrew in the wash-out period following the diagnosis of tachycardia. One subject receiving lignan capsules and another receiving placebo at randomization did not fulfill the FAS because of an eligibility violation (LDL-C<2.9 mmol/L) and were not included in the analyses.

### Baseline Characteristics

The baseline characteristics of participants are given in Table 1. Subjects who agreed to participate were not statistically different from non-participants with regard to age, weight, BMI and gender (data not shown). Of the 68 participants, 60 were taking oral hypoglycemic agents, 46 were taking antihypertensive medications and 5 were taking hypolipidemic agents. The two groups were similar in all observed variables after randomization, except for urinary excretion of isoflavone metabolites and antihypertensive medication usage (data not shown).

**Table 1:** Baseline characteristics of 68 type 2 diabetic patients (full-analysis-set)

Variable	Total (n = 68)	Group A (n = 34)	Group B (n = 34)
Age (years)	63.2±7.4	63.4±7.1	63.0±7.8
Male gender (n, %)	25 (36.8%)	14 (41.2%)	11 (32.4%)
Height (cm)	159.7±8.2	160.5±8.6	158.9±7.8
Weight (kg)	64.2±11.0	64.7±11.4	63.8±10.6
BMI (kg/m^2^)	25.1±3.3	25.0±3.3	25.2±3.3
Waist circumference (cm)	86.9±9.4	87.1±9.6	86.7±9.2
Hip circumference (cm)	94.5±7.0	94.5±7.4	94.5±6.6

Data are mean±Standard Deviation. BMI, body mass index.

### Outcomes and Estimation

No carry-over effect was identified for any of the observed results. Both the lignan capsules and placebo were well-tolerated, and the overall adherence was 96% during the lignan treatment phase and 94% during the placebo phase.

#### Dietary intake and physical activity levels

Pre- post dietary intakes of energy, fat, specific classes of fatty acids, protein, carbohydrate, total fiber and cholesterol are featured according to treatment (Table 2). No significant differences were observed between treatment phases over time. Likewise, no differences were observed for physical activity and other lifestyle practices.

**Table 2:** Dietary intake and physical activity levels of 68 type 2 diabetic patients

Variable	Lignan treatment		Placebo treatment	
	Week 0	Week 12	Week 0	Week 12
Energy (kcal/day)	1911±329	1858±365	1840±327	1866±321
Fat (% of energy)	32±6	31±7	31±6	31±6
SFA (g/d)	10±4	10±4	10±3	10±3
MUFA (g/d)	14±5	14±6	14±5	14±6
PUFA (g/d)	22±9	20±9	21±9	21±9
Protein (% of energy)	17±3	18±3	17±3	17±3
Carbohydrate (% of energy)	51±6	52±7	52±6	51±6
Total fiber (g/d)	10±4	10±3	10±4	9±4
Dietary cholesterol (mg/d)	394±153	439±206	403±195	410±171
Physical activity level(MET-hours/week)	88.0±32.7	92.3±37.7	89.4±32.4	87.6±35.4

Data are mean±Standard Deviation. SFA, saturated fatty acids; MUFA, monounsaturated fatty acids; PUFA, polyunsaturated fatty acids; MET, metabolic equivalent value.

#### Effects on glycemic control and insulin sensitivity

Data of the study endpoints are shown in Table 3. Lignan supplementation significantly reduced HOMA-IR by 3.3% compared to baseline. HbA_1c_, fasting glucose and insulin concentrations were also reduced during the lignan treatment phase, though none of the three pre-post changes reached statistical significance. Compared with the values after 12-week placebo treatment, HbA_1c_ was significantly reduced with lignan supplementation, whereas changes in HOMA-IR, fasting glucose and insulin concentrations were not significantly different between the two treatments.

**Table 3:** Effects of lignan supplemention vs. placebo on glycemic control and lipid profiles in 68 type 2 diabetic patients (full-analysis-set)

Variable	Lignan treatment			Placebo treatment			Treatment difference (*P* value)
	Baseline	12 weeks	Absolute change	Baseline	12 weeks	Absolute change	
Weight (kg)	64.0±11.0	64.5±10.8	0.5±1.3*	64.1±10.9	64.4±11.0	0.3±1.3	0.354
BMI (kg/m^2^) #	25.0±3.3	25.2±3.3	0.2±0.5*	25.1±3.3	25.2±3.5	0.1±0.5	0.478
Systolic BP (mmHg)	139.3±21.4	138.9±19.9	−0.4±15.1	138.0±18.6	138.6±18.2	0.7±11.6	0.268
Diastolic BP (mmHg)	79.2±10.7	77.7±9.5	−1.5±6.7	79.3±10.3	79.0±9.9	−0.3±6.9	0.751
HbA_1c_ (%)#	7.17±1.42	7.06±1.15	−0.10±0.65	7.01±1.10	7.11±1.29	0.09±0.52	0.001
Glucose (mmol/L) #	8.12±2.60	7.83±2.33	−0.29±1.61	7.90±2.31	8.04±2.52	0.14±1.37	0.829
Insulin (µU/mL) #	13.37±4.54	13.08±5.38	−0.29±3.22	13.28±5.02	13.27±4.93	−0.01±2.87	0.169
HOMA-IR#	4.74±1.86	4.49±2.06	−0.25±1.55*	4.59±1.91	4.65±1.88	0.06±1.20	0.142
Cholesterol (mmol/L) #	5.97±0.92	5.81±0.90	−0.17±0.70	5.83±0.85	5.75±0.85	−0.08±0.77	0.367
LDL-C (mmol/L)	4.19±0.89	4.08±0.79	−0.11±0.74	4.10±0.73	4.01±0.76	−0.09±0.71	0.404
HDL-C (mmol/L) #	1.38±0.34	1.36±0.30	−0.02±0.24	1.37±0.32	1.35±0.34	−0.02±0.25	0.243
Triacylglycerol (mmol/L) #	2.25±1.23	2.05±1.10	−0.20±1.12	2.08±1.18	2.11±1.29	−0.01±0.89	0.720
ApoA1 (mg/dL)	152.2±23.2	149.6±23.8	−2.5±20.0	152.4±24.8	148.7±23.9	−3.7±19.7	0.751
ApoB (mg/dL)	111.5±21.2	109.9±21.1	−1.6±16.4	110.3±20.1	108.0±20.5	−2.3±15.4	0.528
Lp(a) (mg/dL)	44.3±37.0 (n = 62)	41.8±34.8 (n = 62)	−2.52±9.85 (n = 62)	43.6±35.3 (n = 62)	43.2±34.7 (n = 59)	−0.59±9.86 (n = 59)	0.339
Urine lignans (µg/mL)#	1.21±2.39	14.20±18.1	13.0±18.2*	1.37±2.50	2.33±7.32	0.97±7.12	<0.001
Urine isoflavones (µg/mL) #	2.70±3.94	2.69±3.67	−0.01±4.56	2.77±4.15	2.66±2.54	−0.11±4.25	0.962

Data are mean±Standard Deviation. BMI, body mass index; BP, blood pressure; HOMA-IR, homeostasis model assessment of insulin resistance; LDL-C, low-density lipoprotein cholesterol; HDL-C, high-density lipoprotein cholesterol; apoA1, apolipoprotein A-1; apoB, apolipoprotein B; Lp(a), lipoprotein(a).

* *P*<0.05 when compared with baseline data.

# Data were not normally distributed. *P* value was calculated using natural-logarithmically transformed data.

#### Effects on lipid profiles

Changes of serum concentrations of total cholesterol, LDL-C, HDL-C, triacylglycerol, Lp(a), apoA1 and apoB did not differ over time or between treatment phases (Table 3).

#### Other effects

As anticipated, the flaxseed lignan supplement significantly increased the urinary excretion of lignan metabolites compared to the baseline and thus validated pill count results during the lignan supplement phase. No difference was seen over time during the placebo phase. Urinary excretion of isoflavone metabolites showed no differences over time or between the treatment phases. Likewise, no differences were observed between treatments for weight, BMI, systolic BP and diastolic BP (Table 3).

### Adverse Events

None of the three participants who withdrew from the study was considered related to the study treatment. Similar numbers of adverse events occurred during both treatment phases and they were predominantly gastrointestinal (percentages of the participants who reported diarrhea, flatulence and nausea were 23%, 32% and 4%, respectively). Blinded reviews conducted on adverse events found that these occurrences were not attributable to the capsules, but rather the participants' health status.

## Discussion

### Interpretation and Overall Evidence

To the best of our knowledge, the present study is the first to investigate the effect of flaxseed-derived lignan on glycemic control, lipid profiles and inflammatory status in type 2 diabetes. We showed that 12-week supplementation of a flaxseed-derived lignan complex, which provided 360 mg/day SDG, statistically significantly reduced HbA_1c_ concentrations in type 2 diabetic patients as compared with the placebo. However, no effect was observed on fasting glucose and insulin concentrations, HOMA-IR and blood lipid profiles.

HbA_1c_, also known as glycosylated hemoglobin, is an indicator of long-term glycemic control over the past six-to-eight weeks, and has been strongly related to micro- and macro-vascular complications in diabetic patients [26]. In the present study, HbA_1c_ concentrations were reduced by 0.10% compared to baseline, which is consistent with a study of dietary supplementation of soy protein (containing 132 mg/d isoflavones) in postmenopausal women with type 2 diabetes (from 6.83±0.64 % to 6.78±0.61%) [27]. Flaxseed lignan (SDG) has been shown to be effective in preventing or delaying the development of diabetes mellitus in animal models, which was thought to be attributable to its strong antioxidant activity [17], [18]. SDG was also shown to suppress the expression of the phosphoenolpyruvate carboxykinase (PEPCK) gene that codes for the rate-limiting enzyme responsible for gluconeogenesis in the liver [28]. Therefore, more studies are needed to clarify the efficiency of lignans in the long-term glycemic control, especially since it is not completely clear whether the relatively small reduction of HbA1c observed in this study is, in fact real, or due to chance.

The lignan capsules used in the present study were specifically selected to avoid potential effects of other nutrients and constituents in flaxseed, such as fiber and α-linolenic acid, which are also highly concentrated in flaxseed. Owing that the maximum safety dose of lignan has not been studied in human subjects thus far, we selected 360 mg/day of SDG, corresponding to 27–60 g of whole flaxseed [20], which was safely used in clinical trials for dyslipidemia [6]–[10]. Consistent with the results of Hallund et al. [14] showing no effect of the flaxseed-derived lignan complex on lipid concentrations in healthy postmenopausal women, we found little effect of pure flaxseed lignan on lipid concentrations in the present study. The cholesterol-lowering effect in previously reported studies [6]–[10] may be due to fiber, α-linolenic acid or other flaxseed constituents, or in combination with SDG, since whole or defatted flaxseeds were used in these studies. Whole flaxseed contains 28% dietary fiber [29], which has been shown to reduce total cholesterol and LDL-C concentrations [30]. Studies using other phytoestrogens (e.g. isoflavones) also found different impacts when isoflavones-containing soy protein was compared to isolated isoflavones. A meta-analysis of 38 clinical trials showed that soy protein consumption significantly decreased total cholesterol and LDL-C concentrations [31]. On the other hand, another meta-analysis found that soy protein with intact isoflavones improved lipid profiles, while extracted soy isoflavones had no effect [32]. A recent study also found neutral effects on blood lipids of isoflavone supplement (132 mg/day) in type 2 diabetic patients [33]. Therefore, it seems that, analogous to soy protein, other component(s) in flaxseed might be required to achieve a therapeutic cholesterol-lowering effect.

The major class of phytoestrogens in Asian diets are isoflavones from soy-rich foods, while in Western diets it is lignan from various plant-origin foods [19]. Supporting this notion, we found comparatively higher urinary excretion of isoflavones than lignans at baseline, which is consistent with a previous study of women in Shanghai [34]. Lignans and isoflavones as two major classes of phytoestrogens are metabolized by intestinal microflora before absorption [11]. However, it remains unclear whether they compete against each other in terms of their absorption and metabolism. In our study, the subjects maintained their habitual intake of soy or soy-products and the lignan supplement did not affect urinary excretion of isoflavone metabolites. The urinary excretion of lignan metabolites (14.20±18.1 µg/mL, i.e. about 70.95 µmol/day assuming a 1.5-L urine excretion per day) in our study (360 mg/day isolated lignan) is comparable to the results of another intervention trial using an isolated lignan complex (500 mg/day) for 6 weeks in a Western population (94±11 µmol/day) [14]. Therefore, there was no evidence suggesting that the bioavailablity of lignans and isoflavones counteract each other. As the intervention was carried out in diabetic patients, more studies are needed to investigate these effects among pre-diabetic and healthy subjects in the future.

One limitation of the present study is that the randomization was based upon the screening results (LDL-cholesterol) rather than the baseline measurements. However, since the recruitment for eligible participants was completed within 6 weeks, it was assumed that the characteristics of the participants would not vary greatly. Secondly, two interventions were done in the spring and the autumn, and the possible influence of seasonal variations may not be fully adjusted even with the cross-over design. Additionally, the relatively modest changes of HbA_1c_ in the lignan treatment might not be clinically sufficient and could be artifact or the effect of regression towards the mean, even when the baseline values have been incorporated into the statistical models.

### Generalizability

In agreement with previous studies [14], [33], we found that a 12-week supplement of a flaxseed-derived lignan providing 360 mg/day of SDG did not affect blood lipid profiles, insulin resistance, fasting glucose and insulin concentrations in type 2 diabetic patients. However, statistically significant reductions in HbA_1c_ were observed with the lignan treatment, though the magnitude of improvement may not be clinically meaningful. Given overall beneficial effects of whole flaxseed consumption for CVD [29] and wide distribution of dietary lignans in plant-based foods, especially flaxseed, there is considerable potential to use these whole foods as adjuvant therapies in type 2 diabetes without apparent side effects. Meanwhile, further studies are needed to confirm the current findings in subjects with different metabolic profiles and/or different ethnic backgrounds.

## Supporting Information

Checklist S1 CONSORT Checklist
(0.05 MB DOC)

Protocol S1 Trial Protocol
(1.28 MB PDF)
