# Supplementary material for: Effects of a Flaxseed-Derived Lignan Supplement in Type 2 Diabetic Patients: A Randomized, Double-Blind, Cross-Over Trial
Source: PLoS One. 2007 Nov 7;2(11):e1148. doi: 10.1371/journal.pone.0001148 (PMC2048577; doi:10.1371/journal.pone.0001148)
Supplement: Protocol S1 — Trial Protocol (1.28 MB PDF) [file pone.0001148.s002.pdf]

内部资料，限相关人员使用，请勿外传

亚麻子健康改善项目

—植物木酚素对 2 型糖尿病患者代谢的影响和机制研究

操作手册

上海市科学技术委员会项目

中国科学院上海生命科学研究院营养科学研究所编制

2006 年 1 月

目　　录

"亚麻子健康改善项目"项目执行组成员名单:..V

"亚麻子健康改善项目"项目办公室联系方式:..VI

"亚麻子健康改善项目"专家委员会（兼质量控制组）成员......................VI

第一部分、项目简介 1

第二部分、研究背景和目的 1

一、研究背景..1
二、研究目的..2

第三部分、试验设计： 2

一、试验设计类型..2
二、随机化分组方法及设盲水平..4
三、研究对象..4
四、样本量计算..6
五、编号..6
六、试验材料..6
七、数据收集..9
八、随访和受试者依从性..10
九、中止和结束试验规定..11
十、不良事件的记录..11
十一、统计分析...12
十二、数据管理和资料的保存..13
十三、试验结束后的随访措施..16

第四部分、受试者筛选 17

一、受试者筛选流程..17
二、受试者筛选总体要求..17
三、筛选问卷..18
四、《受试者筛选表》填写说明..22
五、筛选体检表...23

第五部分、病例报告表 24

一、病例报告表...24
二、《病例报告表》填写说明..34

第六部分、问卷调查 37

一、基线一般调查问卷 37
二、《基线一般调查问卷》填写说明 47
三、三天膳食记录表格 53
四、三天膳食记录表格填写说明 55

第七部分、体格检查标准操作规程 56

一、身高 56
二、体重 57
三、腰围 59
四、臀围 59
五、血压、脉搏 59

第八部分、血样采集标准操作规程 62

一、准备工作 62
二、血样的采集及处理 63
三、血样采集过程的安全事项 67

第九部分、尿样收集标准操作流程 69

一、准备工作 69
二、尿样的采集和处理 69
三、尿样采集过程的安全事项 70

第十部分、质量控制 72

一、问卷质量控制 72
二、体检质量控制 72
三、血样采集的质量控制 73
四、尿样采集的质量控制 73

第十一部分、伦理学问题 74

第十二部分、试验进度表 76

第十三部分、各方承担的职责及其它规定 76

第十四部分、参考文献 77

附录 1 《亚麻子健康改善项目》项目承诺书 80

附录 2 知情同意书·知情告知页 81

附录 3 体检现场组织标准操作规程 84
一、体检前通知与准备 **84**
二、体检方案 **84**
三、工作流程 **85**
四、体检表、胶囊、血样和尿样转运 **85**
五、体检及实验室结果反馈 **85**
附录 4 随访现场组织标准操作规程 86
一、随访前通知与准备 **86**
二、随访方案 **86**
三、工作流程 **86**
附录五 体检表 87

“亚麻子健康改善项目”项目执行组成员名单：

中国科学院上海生命科学研究院营养科学研究所：

林　旭　博士，研究员，　负责项目的总体设计、组织、执行

刘　勇　博士，研究员，　负责项目的总体设计、组织

王燕芳　博士，客座研究员　负责项目的总体设计

于志杰　博士，副研究员，　负责问卷设计和数据分析

黎怀星　博士，副研究员，　负责实验方法建立和实验分析

顾文佳　医学硕士，研究助理，　负责研究项目日常管理和实验分析

潘　安　研究生，　具体负责项目的设计、实施、实验室分析和数据输入和分析

叶兴旺　研究生，　参与体检和问卷调查，实验室分析和统计分析等

孙　亮　研究生，　参与体检和问卷调查，实验室分析和数据输入等

王　婧　研究生，　参与体检和问卷调查，实验室分析和数据输入等

本项目华东医院负责人：

孙建琴　医学学士/公共卫生硕士，主任医师，教授，　华东医院营养科主任　负责指导膳食问卷的设计、录入和分析，受试者筛选，现场体检工作的组织和管理，受试者随访及日常管理

陈霞飞　医学学士，主任营养师，　华东医院营养科　临床研究顾问

孙　皎　医学学士，医师，　华东医院内分泌科副主任　参与临床干预

陈艳秋　医学学士，主治医师，　华东医院营养科　负责膳食调查、录入、处理和分析

宗　敏　医学学士，主治医师，　华东医院营养科　参与临床干预

陈　敏　医学学士，主治医师，　华东医院营养科　参与临床干预

冯　颖　医学硕士，副主任医师，　华东医院营养科　参与临床研究

张鑫毅　医学研究生，　华东医院营养科　参与临床研究

汤丽新　医学学士，主管营养师，　华东医院营养科　参与临床研究

韩维嘉　医学学士，主管营养师，　华东医院营养科　膳食指导宣教

范　青　医学学士，主管营养师，　华东医院营养科　膳食指导宣教

李惠芬　营养师，　华东医院营养科　参与临床干预，受试者随访等

“亚麻子健康改善项目”项目办公室联系方式:

中科院上海营养所

联系人：潘安

E-mail：**apan@sibs.ac.cn**

电　话：021-54920712 或 54920910

地　址：上海市太原路 294 号中科院营养科学研究所

邮　编：200031

上海华东医院

联系人：李惠芬

电　话：021-62483180 转 60406 或 62103

地　址：上海市延安西路 221 号上海华东医院东楼 21 层营养科

邮　编：200040

“亚麻子健康改善项目”专家委员会（兼质量控制组）成员

中科院上海营养所：林旭 博士，刘勇 博士， 王燕芳 博士， 于志杰 博士， 黎怀星 博士；

上海华东医院： 孙建琴 主任医师， 陈霞飞 主任营养师， 陈艳秋 主治医师；

秘书： 潘安

第一部分、项目简介

本项目是由上海市科学技术委员会资助，旨在研究富含植物多酚类物质的亚麻子木酚素胶囊对 2 型糖尿病糖脂代谢的影响和作用机制。中国科学院营养科学研究所将负责本项目的总体实验设计、工作人员培训、一般问卷和血尿样的收集、临床试验用胶囊的发放、实验室生化分析、数据处理、质量控制以及日常工作管理。根据协议，华东医院营养科将负责受试者的招募、受试者的营养教育、体检、面对面随访跟踪记录、三天膳食记录的录入和分析。上海市科委将对实验设计及整个项目的执行过程进行监督。

第二部分、研究背景和目的

一、研究背景

近十年来，随着中国的社会经济的快速发展和居民生活水平的提高，我国居民的膳食结构、生活方式发生了巨大的变化，高血压，糖尿病，高血脂，超重和肥胖等慢性非传染疾病患病率迅速上升。第四次“中国居民营养和健康状况调查”表明，我国成人血脂异常患病率为 18.6%，估计全国血脂异常现患人数 1.6 亿。目前我国 18 岁及以上居民糖尿病患病率为 2.6%，空腹血糖受损率为 1.9%。估计全国糖尿病现患病人数 2000 多万，另有近 2000 万人空腹血糖受损。而且，糖尿病患病率有随着年龄增加而上升的趋势。2 型糖尿病在目前是一种无法治愈的慢性消耗性疾病，不仅极大的影响了患者的生活质量和劳动生产力，而且也消耗了巨额的医疗费用，给个人、家庭和社会造成了巨大的负担。因此，寻找一种经济的、有效的方法用于我国糖尿病的预防和治疗，已成为当今医学和营养科学界的一项紧迫课题。

2 型糖尿病和血脂异常的发生和发展与营养失衡有着密切的关系，同时血脂异常极大的增加了糖尿病患者并发症的危险性，因而通过改善血脂水平来加强对糖尿病控制，改善糖尿病患者生活质量是非常重要的方面。

异黄酮和木酚素是两类最具代表性的植物多酚类物质，前者主要存在于大豆中，而后者则广泛存在于各种植物中，但在亚麻子中含量最高(1,2)。流行病学研究表明，血液中木酚素浓度高的人群其心血管疾病危险性相对要低(3,4)。大豆以及大豆异黄酮摄入量高的亚洲人群其心血管疾病(5,6)、癌症(7,8)等慢性疾病发病率要远远低于摄入量低的欧美人群。欧美国家进行的一系列的临床试验发现，亚麻子可以显著的降低健康人群和高血脂患者的总胆固醇和低密度脂蛋白的水平，从而降低了心血管疾病的危险性(9-16)。而同属植物多酚类物质的大豆异黄酮也在临床上被证实对血脂水平有改善作用，能够降低血胆固醇和甘油三酯、低密度脂蛋白(17-19)。此外，大豆及其异黄酮可显著降低肥胖的 2 型糖尿病患者餐后血糖，改善葡萄糖耐量和糖化血红蛋白水平(20)。大豆异黄酮可阻

止葡萄糖诱导的脂质过氧化，减少小肠对葡萄糖的吸收从而达到降低血糖的作用(21)。动物实验也表明，亚麻子以及其中富含的 SDG（木酚素的前体）可以显著的延缓 1 型和 2 型糖尿病的发生，提高血糖控制，降低总胆固醇等高血脂的危险性(22-24)。然而，迄今为止国内外亚麻子及其衍生物在临床糖尿病预防和控制方面的研究尚未见报道。英国的一项研究发现 50g 从亚麻子中提取的碳水化合物或 23g 亚麻子胶可使健康女性的餐后血糖降低 27％(25)。加拿大的一项研究也发现，高血脂的绝经后妇女服用 40g 亚麻子 2 个月可以使空腹血糖和胰岛素分别降低 5.3%和 10.7%(26)。而且，由于亚洲人群特殊的遗传背景和膳食结构，亚麻子是否对血脂有相应的改善作用也有待研究。

综上所述，本课题拟在糖尿病教育的基础上添加亚麻子植物木酚素补充剂，在国际上首次研究富含植物木酚素的亚麻子对于 2 型糖尿病的血糖和血脂代谢的影响，进一步增进我们对膳食营养抗糖尿病和血脂异常机制的理解。国际上誉为“功能性食物“的亚麻子其主要成分之一—植物木酚素的含量为植物中之冠（比其它的植物高出 75-800 倍），而植物木酚素又广泛存在于各类蔬菜、水果、谷物、坚果和豆类中，因此这一研究工作将为我们今后合理组织膳食来防治目前正在迅速增加中的糖尿病和血脂异常提供科学依据，同时对生物抗糖尿病和血脂异常新药和相关保健食品的开发也具有极其重要的意义。

二、研究目的

系统的研究富含植物多酚类物质（木酚素）的亚麻子对 2 型糖尿病代谢的影响。首要研究问题为：与安慰剂胶囊相比，每天 360mg 的植物木酚素对 2 型糖尿病患者的低密度脂蛋白是否具有改善作用。其次探讨在临床上亚麻子木酚素对于 2 型糖尿病代谢的有关作用机制。具体预期目标包括：

- 研究亚麻子木酚素对 2 型糖尿病病人的脂质代谢的影响及其作用机制；
- 研究亚麻子木酚素对 2 型糖尿病病人的血糖控制和胰岛素敏感性的影响及其作用机制；
- 研究亚麻子木酚素对 2 型糖尿病病人的氧化应激水平的影响及其作用机制；
- 研究亚麻子木酚素对 2 型糖尿病病人的细胞因子及心血管疾病危险因子的影响及其作用机制；
- 研究亚麻子木酚素对 2 型糖尿病病人的体内性激素水平的影响及其作用机制；
- 研究亚麻子木酚素对中老年人的骨质代谢的影响及其作用机制。

第三部分、试验设计：

一、试验设计类型

本课题为随机化双盲临床干预试验，采用交叉试验设计。分两个试验组：一组，先服用植物木酚素胶囊后服用安慰剂胶囊；另一个组，先服用安慰剂胶囊后服用植物木酚素胶囊，但是服用的顺

序和组别对所有的受试者和研究人员保密。试验过程如下图所示：

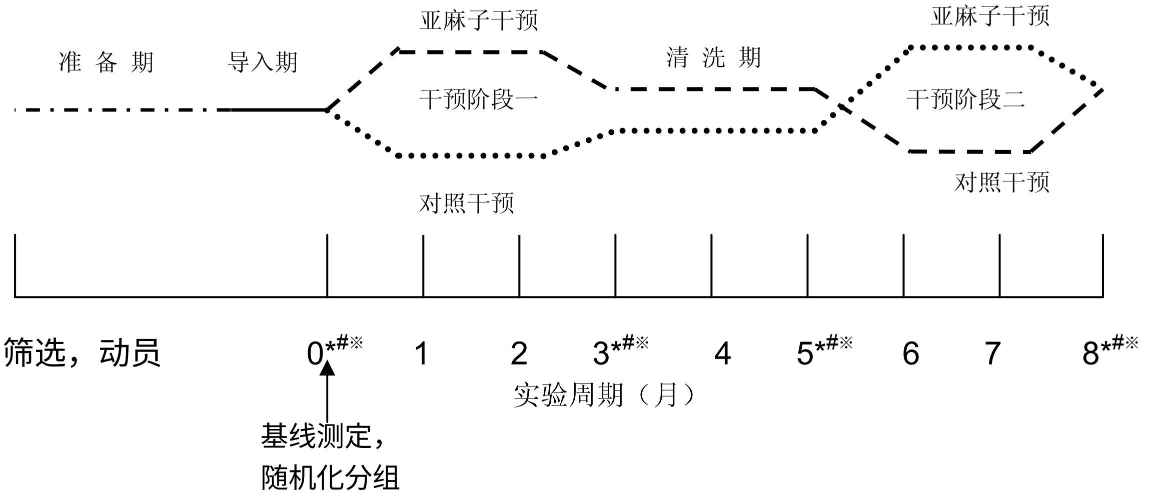

*　血液收集　　#　清晨随机尿样收集　　※　3 天膳食记录和体格检查

图 3－1 试验流程图

试验主要分五个阶段：

（1）准备期（2 月初至 3 月底）：本阶段将进行筛选受试者工作。将按照试验设定的入选和排除标准先对潜在受试者（确诊的 2 型糖尿病患者）进行筛选问卷调查，符合筛选问卷调查者再进行体检和理化指标检查，符合条件者最终入选试验。试验预期样本量为 84 名自愿参加的受试者。

（2）导入期（4 月初）：导入期为一个星期。本阶段受试者将接受项目具体过程的介绍和要求，知情同意书的签署和随机化分组，导入期结束后将进行基线测定，并完成一份一般问卷包括其基本信息、体力活动和豆制品等食物摄入频率，同时记录一份 3 天膳食表格。以上这些是为了对受试者的身体状况、膳食结构和能量摄入进行分析，方便随后进行的随机化分组后的评价；此外，还将对受试者进行体格检查、采血和尿样收集，现场测定空腹血糖水平。

（3）干预阶段一（4 月初至 6 月底）：其中一组将接受亚麻子植物木酚素胶囊干预，另一组将接受安慰剂胶囊干预，本阶段为 12 个星期。此阶段内，至少每周对受试者电话随访一次，所有受试者将被要求前往华东医院进行 3 次面对面随访（每三周一次），及时和研究人员联系反映其病情变化、合并用药和不良事件等。此阶段结束后，受试者将进行体检、血样和尿样的采集，并完成一份简化的一般问卷，记录一次 3 天膳食。

（4）清洗期（7 月初至 8 月底）：本阶段所有受试者都停止试验胶囊的服用，并按照原有的自己的生活方式生活。本阶段内至少每两周对受试者电话随访一次。

（5）干预阶段二（9 月初至 11 月底）：本阶段所有受试者将服用另一种胶囊（和第一阶段相反的胶囊），时间也为 12 个星期。开始和结束时也将有体格检查，血样和尿样的采集，3 天膳食的记录

以及简化的一般问卷调查。

两个试验组在整个试验中除接受的干预胶囊的次序不一样，其他所有教育和干预都是同等的。

二、随机化分组方法及设盲水平

当全部筛选结束后，合格的志愿者被随机化后进入两个试验组。随机分组将采用计算机程序，或使用其他特殊随机方法。参加这项研究的受试者分别有 50%的可能性被分入这两个不同的干预组。研究者和受试者都无法事先知道和选择任何哪一个试验组。

在试验进程中，此项研究项目采取双盲法。随机化之后，每个受试者都有相应的编号，每个编号都对应于一种干预顺序。整个随机过程和随机安排表将由不参与试验的专门统计人员执行和保存。受试者的组别和顺序将对受试者本人、参加干预及数据收集的临床医务人员、数据录入及分析者保密，但试验开始和试验结束时的各项血糖、血脂及血压等生化指标将及时告知受试者。仅在特殊情况下，如受试者由于其他合并疾病的治疗需要知道接受的干预方式时，将根据一定的程序告知此受试者接受的干预方式。

在整个干预试验和实验室指标分析的过程中，受试者的组别和顺序对受试者和研究人员保密。实验室分析结束之后，统计分析之前，研究人员将被告知两组受试者的名单，但是两组接受的干预方式仍将保密。整个统计分析结束之后，受试者和研究人员才被告知每个受试者接受的干预方式，即整个盲底的揭晓。

三、研究对象

生活在上海市区，确诊患有 2 型糖尿病的志愿者。年龄在 50-79 岁（1927 年 1 月 1 日－1956 年 3 月 1 日出生）。预期招募 67 人，男、女性皆有。

（一）纳入标准

- 不使用胰岛素对其血糖进行控制；
- 年龄为 50-79 周岁；
- 高血脂：低密度脂蛋白（LDL）≥2.9mmol/l(111mg/dl)；
- 女性要求阴道停止出血至少 1 年；
- 目前或试验前 3 个月没有参加其他研究项目；
- 具有良好的记忆和活动能力；
- 生活能够自理；
- 能够自行签署知情同意书；

（二）排除标准

- 除了糖尿病以外的任何其他原因导致的高血糖症（如原发性高血糖症）；
- 目前或曾经（试验之前 6 个月内）使用了雌激素疗法或者固醇类激素疗法；口服或者肠道外注射用皮质固醇类；
- 经常服用富含异黄酮或木酚素等植物多酚类物质的补充剂（试验之前 3 个月内）；
- 急慢性感染；经常性使用抗生素等影响实验者（经常性使用定义为试验前 3 个月平均每个月使用 2 个疗程或以上）；
- 使用毒品或者酗酒者（经常喝酒定义为女性摄入酒精>40 克/天，约为 250ml 黄酒，或 400ml 啤酒，或 80ml（2 两）白酒，男性摄入酒精>80 克/天）；
- 癌症患者或五年之内接受过放射治疗或化学治疗患者；
- 严重肝、肾功能疾病患者（谷丙转氨酶、血清肌酐指标高出华东医院设定的正常值范围 1.5 倍以上者）；
- 活动性消化道溃疡或者影响食物消化吸收的其他疾病（如长期腹泻，消化不良等）；
- 严重甲状腺疾病（甲亢或甲退）或垂体疾病；
- 收缩压≥180 毫米汞柱或舒张压≥120 毫米汞柱，或III期高血压病患者；
- 任何精神疾病；癫痫患者或者使用抗癫痫治疗中；使用抗抑郁药物者；
- 目前或曾经（6 个月内）有过严重心脑血管疾病，可能会干扰试验的正常进行（如心衰、6 个月内有过心肌梗塞，脑梗塞，急性心肌炎，严重心律不齐，接受介入式疗法者等）；

（三）退出试验标准

- 发生严重不良事件；
- 试验对象未能按照规定剂量、次数等服用亚麻子木酚素胶囊连续 7 天以上；
- 发生其他可能影响病人治疗结果的疾病；
- 服用了本研究禁止服用的药物；
- 失随访；
- 研究对象要求退出；
- 主要研究者认为研究对象有必要终止本项研究；

（四）招募受试者及筛选合格受试者

由华东医院及其它医疗机构的帮助下，筛选合格的受试者。由营养所和华东医院的有关人员通过项目介绍和发放有关宣传资料使可能的受试者了解研究目的及入选条件。在签署知情同意书之后，将符合标准者请至医院门诊进行问卷和体检，并采集血样进行有关的生化指标分析（空腹血糖、

胰岛素、总胆固醇、甘油三脂、低密度脂蛋白、高密度脂蛋白、血清肌酐、谷丙转氨酶、血常规)。对所有合格者进行随机化分组确定干预方式。

四、样本量计算

样本含量计算公式：本试验为交叉试验设计，采用配对的样本量计算公式：

$$N_d = \frac{(Z_\alpha + Z_\beta)^2 \sigma^2_d}{\delta^2_d}$$

其中N_d是配对的样本数；σ_d^2为配对数据差值的的方差；δ_d为配对数据的差值。

使用单侧显著性水平为 5%时，设定把握度为 90%，根据估计低密度脂蛋白的平均水平(3.4mM)，根据以往类似的临床试验结果，设定配对的低密度脂蛋白差别为 0.2mM（6%的改变量），标准差为 0.5mM，计算所得样本量为 54 人。并考虑可能的退出者（约 10%的脱落率），$N^*=N_d/(1-R_0)^2=54/(1-0.1)^2=67$，最终样本量共为 67 人。

五、编号

（一）编号原则和方法

受试对象每人有一个固定编号为 3 位数，从 001 开始。如“054”表示第 54 号受试者。其中编号排列无固定次序，由电脑随机产生。

但每位受试者还有另外若干个编号为四位数，其中后三位数字为其固定编号，第一位数字表示其体检和问卷次序，中间以横线“-”隔开。本试验共有四次体检，四次膳食记录，四次一般问卷调查。所以最前一位数字为“1”表示第一次体检或问卷等，“2”表示第二次，“3”表示第三次，“4”表示第四次。如“2-054”表示第 54 号受试者第 2 次体检或问卷等信息资料。

（二）编号条使用

三位数字固定编号用于病例报告表，试验胶囊标签，受试者手册等各项本试验中只使用一次的表格等材料上。

四位数字编号用于各次体检表，问卷，采血和分血管，尿样保存瓶，采血袋，问卷袋等各项本试验中数次使用的材料上，第一位数字“1”、“2”、“3”、“4”表示次序。

六、试验材料

试验用胶囊的理化性质：

本试验中每位受试者都将接受亚麻子木酚素胶囊和淀粉安慰剂胶囊的干预(即前后两个干预阶

段的干预胶囊不同），两种胶囊在外观、重量、大小、气味等方面基本一致，以保证盲性。正常天然的亚麻子由坚硬的外壳和里面的种子两部分组成，木酚素和纤维主要存在于外壳中，而种子中主要含有油料成份。本试验将利用酒精提纯过的亚麻子粉末来制作亚麻子胶囊，从而排除其中含有的油料成分（主要为ω-3 脂肪酸）和膳食纤维的影响，以研究木酚素对于代谢的影响。每天需服用 1.8 克亚麻子胶囊（共 3 颗）。安慰剂胶囊采用米粉为主要原料，基本无副作用，作为安慰剂广泛应用于临床试验中。

胶囊将按照标准保健品生产程序生产，每颗胶囊重 600 毫克，其中含木酚素 120 毫克左右，基本浓度为 20%。安慰剂采用标准的淀粉胶囊，同样每颗胶囊重约 600 毫克。

给药途径：口服，温水送服。

给药次数：第一次收到胶囊的一个星期内（4 月初和 9 月初的时候），采取渐进式的服用方式。第一天服用 1 颗胶囊，如没有任何明显的副作用，第二和第三天服用 2 颗胶囊；如没有任何明显的副作用，则从第四天开始每天服用 3 颗胶囊并保持下去。如在以上步骤中任何一步有明显的副作用，则停止服用胶囊并立刻和研究人员联系，由研究人员根据实际情况采取相应的措施。

服用时间：每天早、中、晚共三次，每次一颗。饭前和饭后服用均可。

试验用胶囊的编码和登记：

对试验胶囊的编码将由负责随机化的专人执行。在对受试者接受的胶囊进行编码时，先列出两种胶囊的随机安排表（即接受该胶囊的受试者编号），然后取出一种胶囊如 A 胶囊分装至给予受试者的小瓶中，分别依次在瓶上只写上该病人的编号，不标记是何种胶囊。A 胶囊分装完毕之后，再对 B 胶囊进行分装。研究人员得到的只是带有受试者编号的瓶子，研究人员根据上面的编号把胶囊发至相应的受试者手中。因此，研究人员和受试者得到的只是带有受试者编号的瓶子，并未知道里面装有何种胶囊，从而保证了双盲的顺利进行。

干预阶段一时间为 12 个星期，分四次发给受试者胶囊，分别为试验开始及之后每三周一次（即每次面对面随访时）。受试者将被要求在领取胶囊时登记领取胶囊的数量和时间，并返还上一周期未服用完的胶囊及其瓶子，如表 3-1 所示（附于病例报告表中）。干预阶段二发放胶囊方法和阶段一是一样的。

表 3-1 胶囊的发放和回收

编号	发放数量	回收数量	丢失数量	负责医师签字
	□瓶，共计□□颗	□□颗	□□颗	

注：由于第一次发放胶囊时并无回收和丢失，因而在第一次时没有这两项。

试验用胶囊的贮存和注意事项：

（1）贮存于阴暗干燥室温，避免阳光直射（天热可以放冰箱中）。

（2）请将此胶囊放在儿童不能接触的地方。

（3）孕妇或计划怀孕者慎用。对亚麻子过敏者慎用。

（4）每天服用 3 颗胶囊，请勿多服。

试验用胶囊的规定：

本批胶囊只可用于本次临床研究项目，不得用于其它目的，更不得用于商业和销售市场。如有违反，可由主要研究者追究此人的法律责任。

合并用药的规定：

合并用药需要既不影响对试验胶囊疗效和安全性的观察，又不能影响病人必须用于其他病症的治疗。受试者在试验过程中服用了不应当服用的合并用药，常常是被剔除或排除出分析集的原因之一。在本试验中，主要是研究亚麻子对于 2 型糖尿病人血脂、血糖、胰岛素以及炎性因子和氧化应激水平的影响，但亚麻子为一种天然谷物，不能代替受试者的药物治疗。根据这个原则，特制定了以下的规定：

（1）受试者保持原有的药物使用和治疗方式；如若改变（包括剂量和种类等），请立即通知研究人员，并如实登记在"合并用药表"上，如表 3-2 所示。

（2）未经研究人员允许，禁止接受以下治疗或服用以下药物或保健品：激素疗法，激素或固醇类药物，非甾类抗炎免疫药，抗生素，未经许可的中药、中成药或天然药，富含植物多酚类物质的保健品（如天然元、大豆异黄酮、葛根素、木酚素、女人馨等）。

（3）服用胶囊时，温水送服，避免用茶、咖啡等送服。服用胶囊时间为饭前或饭后。

（4）任何身体不适或需要治疗时，请事先征求研究人员意见或事后立即通知研究人员。

表 3-2 合并用药表

	伴随用药（请填写通用名）				用药目的	开始日	终止日
	名称	剂量	频率	给药途径			
1				□1 口服<br>□2 肌内注射<br>□3 静脉点滴<br>□4 静脉推注<br>□5 其他<br>( )	□1 治疗自身疾病<br>□2 治疗不良事件<br>(AE 号)<br>□3 其他<br>( )	2006 年<br>□□月<br>□□日	2006 年<br>□□月<br>□□日
2				□1 口服<br>□2 肌内注射<br>□3 静脉点滴<br>□4 静脉推注<br>□5 其他<br>( )	□1 治疗自身疾病<br>□2 治疗不良事件<br>(AE 号)<br>□3 其他<br>( )	2006 年<br>□□月<br>□□日	2006 年<br>□□月<br>□□日

可能的副作用：

亚麻子为天然食物，口感和安全性都很好，其提取物植物木酚素为纯天然制剂，无国家禁止的任何添加剂，未发现明显的副作用，但极少数个体也有可能会出现食物过敏：如皮疹；皮肤瘙痒；面颈部潮红；胃肠胀气；腹泻；恶心；呕吐；大便次数增加等。

七、数据收集

（一）数据收集

以面对面随访的形式收集所有的问卷资料。在规定时间，通知受访对象到指定的地点进行体检和血样采集，具体内容见表 3-3。

表 3-3 数据收集内容

	内容	简要描述
一般问卷	人口统计学方面	年龄、性别、婚姻状态、职业、出生日期
	社会经济状态	教育的年限，个人和家庭的收入
	病史，糖尿病管理	包括糖尿病和高血脂在内的所有健康问题，以及药物治疗情况
	吸烟和饮酒	目前吸烟和饮酒的状况：包括数量、类型等
	体力活动水平	包括工作和休闲时间在内的体力活动和体育锻炼情况
膳食问卷	膳食调查	3 天膳食记录和简单食物频率问卷
体格检查	体格测量	身高、体重、腰围、臀围
	体格检查	血压、脉搏
血样采集和分析	血样采集	红细胞、白细胞、血浆和血清样本
	尿样采集	清晨空腹随机尿收集
	现场血样分析	空腹血糖

注：以上数据收集和体检均由参加统一培训并经考核合格的调查者执行，整个过程均有严格的质量控制。

整个试验过程中将有四次血样和尿样采集，分别为两个干预阶段的开始和结束时。

血样采集每次为 10 毫升，加入 EDTA 抗凝剂（紫色管）和促凝剂（红色管）各一管，5 毫升/管，分别得到血浆和血清样本。含 EDTA 的血样管必须在抽血后 15 分钟内进行离心分离，然后将血浆、白细胞和红细胞分别分装入不同的冻存管内，最后放入-20°C的冰箱内暂时保存并于当天转运至-80°C冰箱内保存。不含 EDTA 的血样管静置于室温 30 分钟后离心，然后将血清分装入不同的冻存管内，最后放入-20°C的冰箱内暂时保存并于当天转运至-80°C冰箱内保存。

尿样由受试者在家中采集于一次性塑料杯中，然后倒至专门的尿样保存瓶中，带至体检现场。研究人员接到尿样保存瓶后，加入 50 毫克抗坏血酸（防腐剂），充分摇匀后，分别量取 1 毫升至 6

个小的尿样保存管中，最后都放入-20℃的冰箱内暂时保存并于当天转运至营养所-20℃冰箱保存。

整个试验过程中将有四次一般问卷调查（包括一般信息，体力活动和豆制品等食物频率），分别为两个干预阶段的开始和结束时。将有四次 3 天膳食记录，分别为每次体检前一周内的三天，必须包括两个工作日和一个周末。

体格检查结果将在每次体检后及时反馈给受试者，血液和尿液指标分析将在整个临床干预结束后在中科院营养所内采用国际通用的方法测定，血糖、血脂、胰岛素、糖化血红蛋白等常规指标将在测定完成后反馈给受试者。

（二）临床和实验室检测项目

常规代谢产物：空腹血糖，HbA1c，C－肽，血脂（总胆固醇、低密度脂蛋白、高密度脂蛋白和甘油三脂）分析等；

氧化应激/抗氧化指标：MDA，SOD，isoprostane，GPx，GSH/GSSG，protein carbonyl 等；

激素： insulin，Adipokines（leptin, adiponectin），IGF-1，TNF-α；

细胞因子：Cytokines（ IL-6, Eta-1），CRP，PAI-1，Chemokines(mcp-1)，SOCS3 等；

性激素：睾丸激素，雌二醇，性激素结合球蛋白等；

植物多酚类物质代谢：血液和尿液中木酚素和异黄酮等植物雌激素类物质浓度；

骨质代谢：碱性磷酸酶，osteocalcin 等；

八、随访和受试者依从性

为随时了解受试者的身体状况和服用胶囊情况，以保证试验的顺利进行和防止严重不良事件的发生，相关研究人员将对受试者每周进行电话随访一次，每三周进行面对面随访一次。随访主要收集受试者在随访前三个星期内的相关信息：服用胶囊情况、不良事件和不良反应、合并用药、生活方式和体力活动等的变化，每三周发放新胶囊和回收未服用的胶囊，监测血糖、血压、体重等。

受试者的依从性主要是通过两种方式来计算：第一，计算回收的胶囊数量。依从性=（发放胶囊数量-回收胶囊数量）/理论服用胶囊数量*100%。依从性达到 80%以上的受试者才能被纳入最终的符合方案集统计分析。第二，分析血液和尿液中木酚素浓度。通过比较试验前和试验后的数值来计算依从性。一般服用异黄酮或木酚素等植物多酚类物质之后，血液中的浓度以及尿液中排出的浓度会增加十几到上百倍的增加（不同的人对植物多酚类物质的代谢吸收不同）。

为保证受试者的依从性，主要采用以上措施：第一，在随访时进行监督和教育。听取受试者主诉，征求受试者意见，根据其生活方式和膳食特点制定切实可行的个性化的服用胶囊计划；第二，平时不定期电话询问受试者的各方面的情况，如服用胶囊情况，对于自身病情的感觉，生活方式和膳食的改变，对于试验的意见等，并耐心的回答受试者对于健康等方面的问题，帮助受试者树立科

学的营养观念，并加强糖尿病和高血脂等疾病防治方面的教育，建立和受试者之间的良好关系。

九、中止和结束试验规定

（一）中止试验标准

- 在试验过程中出现了严重不良反应（危及生命，或影响正常的一般性工作和生活）；
- 半数以上受试者出现中度不良反应；

（二）结束试验标准

- 按照试验预期时间，即干预期为 8 个月。

十、不良事件的记录

不良事件是指临床试验受试者接受该试验胶囊后出现的不良医学事件。需要指出的是，不良事件并不一定与治疗有因果关系。事件发生后，要根据医学知识判断其是否为不良事件。

不良反应是指在按规定剂量正常应用试验胶囊的过程中产生的有害的非预期的、与试验用胶囊有因果关系的反应。因此，不良反应都是不良事件，但不良事件不一定是不良反应。试验中，对于不良事件是否与试验用胶囊有因果关系则是由本临床试验的研究者组成的专业委员会来确定。

严重不良事件与事件严重程度的“严重”不是同一个概念。严重不良事件是指：①死亡；②危及生命；③须住院治疗或延长原来的住院时间，或导致入院；④严重或永久的残疾或失能；⑤严重的医疗事件，指不一定马上危及生命、导致死亡或住院的情况，但是极大的危害了受试者，需要采取措施预防以上严重后果的发生。如果是严重不良事件，务必填写《严重不良事件报告表》。

本试验中对于不良事件的严重程度分类方法以及不良反应的判断方法见《病例报告表》的填写说明（本操作手册第35页）。

受试者被要求在发生不良事件后，立即联系研究人员，征求研究人员对于不良事件的处理意见。研究人员在接到不良事件的报告之后，应立即要求受试者停止服用胶囊，并尽快讨论处理措施，判断该不良事件是否和与试验胶囊有关。如判断为无关，可通知受试者继续服用胶囊，但仍需密切观察；如判断为有关，则因根据严重程度决定减轻剂量或暂时停止该受试者的胶囊服用，直到让病人退出试验。对于发生不良事件的受试者，应密切观察其不良事件和身体健康的发展情况，随时采取措施预防严重不良事件的发生。

发生严重不良事件时，受试者或受试者家属应立即暂停其试验胶囊的服用，并送至附近的医院治疗，尽快通知研究人员。研究人员接到严重不良事件的报告后，应立即前往医院陪同受试者进行诊断和治疗，在 24 小时内通报主要研究人员并尽快召开研究人员会议，并在 48 小时内通报伦理委员会。组织独立的医学专家委员会，由专家委员会鉴定严重不良事件是否与试验胶囊有关。如若

有关，应立即中止试验，并通知所有受试者停止服用胶囊。研究人员将根据医学专家委员会的鉴定结果对与试验有关的损害提供治疗费用和相应的经济补偿。

任何时候，受试者发生急诊需要就医时，应停止服用胶囊，并立即前往就近医院治疗，而后通知研究人员。

十一、统计分析

本试验的首要目标是研究亚麻子对于 2 型糖尿病人中低密度脂蛋白水平的影响。次要目标是研究亚麻子对于 2 型糖尿病人中其他血脂代谢、血糖、胰岛素、糖化血红蛋白以及炎性因子、性激素水平、氧化应激水平等的影响。血糖、血脂、糖化血红蛋白等常规指标将采用日立自动生化分析仪测定，胰岛素、炎性因子等将采用 Luminex 仪器测定，性激素指标将采用放免的方法测定，氧化应激水平指标将采用 ELISA 的方法测定。因此，主要根据试验四次血样和尿样的这些生化指标的结果，来验证亚麻子的疗效。

每个受试者都将接受两个阶段的干预，分别为亚麻子木酚素胶囊干预和安慰剂胶囊的干预，根据每一阶段前后的数据的比较，可以得到每个受试者分别接受木酚素和安慰剂胶囊的引起的变化，定义为试验组和对照组。采用mixed模型比较试验组和对照组的各生化指标的差异，以试验组和对照组的 12 周之后的生化指标结果为因变量，以干预（木酚素或安慰剂）、周期（第一或第二周期）作为固定因子，受试者编号作为随机因子，基线指标结果作为协变量，同时控制干预*周期、干预*基线结果这两个交互项。考虑到试验很多生化指标是体重关联的，因此可以将体重或体重变化作为自变量放入模型中观察影响。两试验组各次体检的结果的比较采用成组t检验，干预前后的计量资料采用配对t检验，计数资料采用频数（构成比）进行统计描述，各组干预前后的变化采用χ^2 检验或非参数检验。临床观察指标用Wilcoxon符号秩检验（Wilcoxon signed-rank test）进行比较。使用多重线性回归来分析血脂和血糖等生化指标变化的影响因素。不是正态分布的数据在统计分析时需经过自然对数转化。

临床试验的统计分析首先要考虑的是统计分析集。在本试验中主要按照以下的原则来进行统计分析。

(一)统计分析的数据选择

- 意向性分析（Intention-to-treat）：将所有经过随机化的受试者作为所分到处理组的病人进行随访、评价和分析，而不管其是否依从计划的干预过程。这对于防止偏性是有益的，但是在实际分析中存在不合理性，因此这仅是一个原则。
- 全分析集（Full analysis set）：对所有经随机化分组，并至少服用一周的全部病例进行分析。对于未能观察到全部干预过程的病例资料，用最后一次随访数据结转到试验的最终结果(last

observation carry forward)。

全分析集剔除病例的原则：（1）违反合法性：受试者违反了主要的入组标准，不应当进行随机化。（2）受试者服用胶囊少于一周。（3）随机化之后没有任何数据。

●符合方案数据分析(Per-Protocol Population)：所有符合试验方案、依从性好（每次随访服用胶囊率达到70%以上，总的实际服用量占应服用量80%-100%）、试验期间未服用禁止药物、没有重大的对试验方案的违反的病例，对其疗效进行统计分析。

●安全集（Safety set）：只要受试者用过一次或以上其所在组的胶囊而不管在服用胶囊以后是否有检查结果的数据都要纳入安全集，就应当研究其不良反应或不良事件。

(二)统计分析计划

统计分析将采用 Stata 统计分析软件进行计算。所有的统计检验均采用双侧检验，p 值小于或等于 0.05 将被认为所检验的差别有统计意义。

● 一般性描述统计

所有测量得到的计量资料采用均数±标准差进行统计描述。与筛选期基础值进行比较，采用配对t检验比较干预前后差异以及对照组和试验组的差异。两试验组各次体检等计量数据采用成组t检验进行比较。两组各次取得的计数资料采用频数（构成比）进行统计描述。各组干预前后的变化采用χ^2检验或非参数检验。多元线性回归将被用作分析血脂和血糖等生化指标变化的影响因素。

✧ 脱落分析：各组总脱落率和主要脱落原因的比较采用χ^2检验。

✧ 基础值的均衡性分析：采用t检验或χ^2检验比较人口学资料和其他基础值指标，以衡量两组均衡性如何。

✧ 有效性分析：采用 mixed 模型评估有效性指标。比较对照组与试验组之间各项测量指标的不同，如血糖、胰岛素、糖化血糖蛋白、血脂、血压及其他相关的指标。

✧ 相关性分析：多元回归统计方法将被用于检验与糖尿病控制有关的指标和膳食中植物多酚类物质摄入的关系，以及影响血糖和血脂等生化指标变化的因素。

十二、数据管理和资料的保存

数据管理包括数据搜集阶段的数据检查核对，数据整理阶段的预处理、编码、录入、校对和清理等。主要是为了净化原始数据，使其系统化、条理化，确保数据质量，确保其正确性和科学性。数据管理主要涉及三方人员：研究人员、质量控制组和数据管理员。

（一）研究人员

研究人员的主要职责之一是将受试者的信息正确、清晰的填入预先设计好的病例报告表和各项问卷，并在表上签名。病例报告表和各项问卷上的数据不能任意修改，如确实需要修改，可将原数

据用两横线划去（要保证原数据依然可见），并在旁边写上正确数据，且需要签署姓名和注明日期，重要数据的修改还须注明修改原因。对于病例报告表和各项问卷上的数据，研究者都应该核实，特别是对于一些异常值，必要时应加以说明。此外，数据管理员在应用处理数据时可能会产生疑问，研究人员应对数据管理员返回的数据疑问表认真核对，并给予答复。

（二）质量控制组

质控组成员要定期前往试验现场，监督各个研究人员的试验进度及病例报告表的填写。在数据送到数据管理部门之前，质控组需要对每一份收到的表格进行一系列的核对，主要是以下几个方面：

（1）总的检查：核对受试者登记及随机化，检查表格是否及时收齐并有研究人员的签字，受试者的编号是否准确等。

（2）入组和排除标准的检查：检查受试者的入组和排除是否遵循试验方案。

（3）缺失数据：是否有些项目没有填写。如有，应立即询问研究人员，并尽可能找到和填上原始数据。

（4）数据的填写是否符合规范：是否按照病例报告表、各种问卷的要求填写数据。

（5）数据范围的核对：检查有些项目数据是否超出范围。

（6）逻辑检查：各项目的回答之间有无逻辑性错误。

（7）检查合并用药、不良事件、干预变更等信息是否都已经被记录或报告。

一旦质控组查出有错，应及时送回研究人员处加以校正。病例报告表和各项问卷只能由研究人员进行修改，研究人员在修改的数据旁应签上自己的名字。最后，经过核查的数据要及时送交到数据管理员。病例报告表、各项问卷等信息的移交、传送都应在相应的表格中有所记录。

（三）数据管理员

数据管理员的主要职责是将病例报告表和各项问卷上的信息准确、可靠的输入计算机，并保证数据库和所有资料的安全和保密。主要有以下工作：

（1）建立数据库。

本试验中主要采用 EpiData 软件进行数据的录入。为了确保数据录入的准确性，采用双份录入的方式。对于 EpiData 不能进行访问权限的控制和不能追踪修改痕迹的缺陷，采用其他的相应的措施进行弥补，如采用各种密码设置软件、定期备份数据文件并作记录等。

（2）病例报告表和各项问卷的交接和检查。

数据管理部门在接到病例报告表和各项问卷之后，首先要进行交接：核对收到的表格是否完整，有无缺页、缺表，编号是否与监查员的移交记录一致。同时，数据管理部门和质控组之间的各项表格的交接也要在相应的数据移交表格中记录。

接到数据后，首先进行目视检查，检查内容和质控组的检查相同。检查中发现的任何问题应及

时通知监查员，由质控组要求研究人员作出回答。数据管理员和研究人员之间的问题可以用疑问表（query form）的形式。疑问表能保证数据的修改都有据可查，避免被人为的任意修改。同时，疑问表发送出去之后应留有备份，以便查询。

（3）建立数据结构。

利用 EpiData 软件建立数据的输入界面，同时产生数据文件结构。输入界面尽可能考虑到病例报告表和各项问卷的安排顺序，也应充分考虑方案的要求、录入和分析的方便，尽可能减少数据输入的错误的发生，以保证数据的完整、正确和安全。

建好的数据文件结构需要经过测试正常后才能使用。将使用预试验的数据和模拟的数据对数据结构进行测试，测试的数据文件应提交数据文件测试报告。任何关于数据文件结构的修改都应有记录，并需要保存修改前的数据文件结构，不能随意删减。

（4）数据录入和校对

数据录入前将对录入人员进行培训，以熟悉数据录入界面和要求，培训合格之后才能进行数据的录入工作。录入采用双份、独立录入方式，即两个录入员用相同的数据分别独立的录入同样的数据文件。EpiData 软件具有对双份输入数据进行比较并打印出不一致部分的功能，不一致的部分将由录入员对照原始的数据分别进行修改。

（5）数据文件的逻辑检查

数据输入中难免有错误产生，因此在数据录入结束后需要对数据进行逻辑检查和清理。计算机处理过程中的逻辑检查包括在数据录入时和录入以后用程序进行的检查。

数据录入时的逻辑检查应该是在数据文件建立时就设置好，并与数据文件结构一同接受测试。EpiData 软件中的 Check 程序可以编制这种逻辑检查文件，主要包括输入范围的校对、逻辑跳转、自动编码、输入警告提示等。

但有些检查和校对无法在数据输入的同时进行（如不同数据库之间的逻辑关系检查等），这可以用相应的软件如 SAS 软件进行。所有的错误内容及修改结果都应有详细记录并妥善保存。如果是需要研究人员回答的问题，需要填写疑问表，由监查员送交相关的研究人员，由研究人员对这些问题进行检查和回答。

为了保证数据的质量，数据录入完成并清理结束后要对数据进行质量抽检。随机抽取数据文件中的 5%的记录，对全部变量或主要变量进行手工比对，检查录入的数据与原始数据是否一致。对于数据的合格，规定为：主要观察指标必须 100%正确，其它数据的正确率在 0.5%左右。如果没有达到这个要求，需要对数据库重新进行清理和检查。

（6）盲态核查和数据锁定

盲态核查（blind review）是在最后一份数据输入后、第一次揭盲之前对数据保持盲态的审核。

以便对各受试者属于什么分析集做出最后决定，并用文件形式记录下来。在盲态审核下作出的决定在揭盲之后不能被修改！

在盲态审核并认为所建立的数据文件正确无误之后，由主要研究者、生物统计学专业人员和保持盲底的有关人员对数据库进行锁定。此后，对数据库的任何改动只有在以上几方人员同意（书面形式）的情况下才能进行。数据库锁定后才可以进行第一次揭盲并移交统计分析。

资料的保存：

所有关于本试验的资料将被保存在中科院营养所，由主要研究者负责资料保存的完整性和安全性。未经主要研究者同意，任何数据库和资料的外泄都属于违规行为。需要使用数据库的人员必须签署“使用资料协议”，以保证数据库和资料的安全性。资料的保存要求和期限将严格按照国家的有关规定执行。

十三、试验结束后的随访措施

在试验结束之后，依然要对受试者进行随访，以观察试验用胶囊是否有滞后或残留作用，对受试者是否有不良影响。随访的方式主要是采用电话随访。随访的主要内容包括受试者对自身健康状况的主诉、是否有不良事件的发生等。发生不良事件后，依然要判断是否与试验用胶囊有关。如确实有关，要采取相应的措施，为受试者检查并推荐专业医师进行治疗，同时密切观察其他受试者是否有类似情况出现。试验结束后的随访期为两个月。

试验结束后，将对受试者进行糖尿病教育和膳食、生活方式的指导，以便受试者以后更好地通过膳食和生活方式的改变来控制自己的糖尿病和高血脂症状，最终提高自身的生活质量。

第四部分、受试者筛选

一、受试者筛选流程

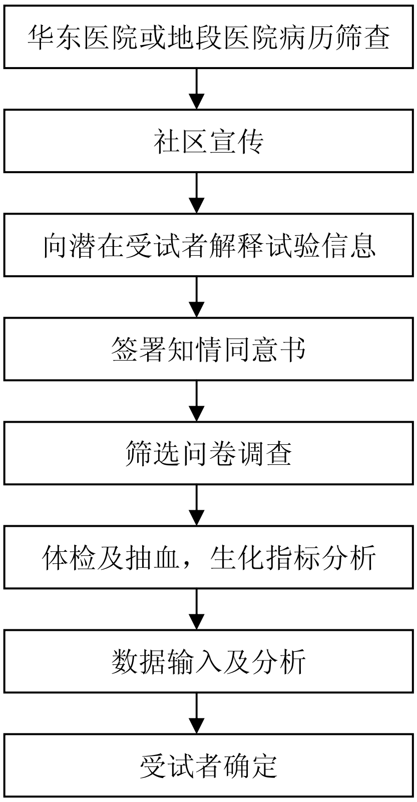

图 4-1 受试者筛选流程图

二、受试者筛选总体要求

1 严格遵守《赫尔辛基宣言》和医学伦理道德，尊重受试者选择，保守受试者信息。

2 严格按照筛选流程进行操作，只有上一步骤结束才可以进入下一步。

3 必须向潜在受试者解释清楚试验情况，待其完全了解并签署知情同意书之后方可进行体检和抽血。

4 体检和抽血要求按照正式试验的标准进行，具体方法和要求见第七部分。体检包括：身高，体重，腰围，臀围，血压。筛选抽血检测指标包括：空腹血糖，血脂（总胆固醇、甘油三脂、低密度脂蛋白、高密度脂蛋白），胰岛素，血常规，血清肌酐，谷丙转氨酶。

5 筛选过程中的每个步骤和工作都应该记录在案，包括问卷筛选和体检筛选不合格者的基本信息以及排除原因，以备审查及重新翻阅。

6 华东医院和中科院营养所应及时沟通，共同完成受试者的筛选工作。

三、筛选问卷

亚麻子健康改善项目

（受试者筛选表）

编号：____________________

姓名：_____________________

住址：_____________________

电话：_____________________

上海市科学技术委员会项目
中国科学院上海生命科学研究院营养科学研究所编制

2006 年 1 月

填表说明：请仔细阅读，并在相应的选项上画“√”或在“□”内填写数字。

1．姓名：________________ 编号：________________

联系方式：________________

2.性别： (1)男 (2)女

2．1 如是女性，请回答最后一次月经什么时候：□□□□年□□月

如记不清楚，请回答是否在 2005 年 3 月 1 日之前？

（1）是 （2）否

2．2 如是女性，请回答最近**六个月内**是否使用了雌激素疗法或服用激素类药物：

（1）是 （2）否 （3）不清楚

3．出生日期：19□□年□□月□□日

4．您是否为 2 型糖尿病患者？

（1）是 （2）否

5．生活是否能够自理？

（1）是 （2）否

6．您过去**三个月内**使用过胰岛素对您的血糖进行控制吗？

（1）是 （2）否

7．您过去**三个月内**是否参加其他研究项目？

（1）是 （2）否

8．您过去**三个月内**是否常规使用抗生素、抗菌素等药物？（常规使用定义为 3 个月内平均每个月使用一个星期或以上）

（1）是 （2）否 （3）不清楚

如是，请回答使用的原因：________________ 剂量：________________

9．过去**三个月内**是否常规使用阿司匹林等水杨酸类药物？

（1）是 （2）否 （3）不清楚

如是：剂量如何：________________

10．五年之内是否有过放疗或化疗？

（1）是 （2）否 （3）不清楚

11．您是否饮酒？

（1）是　　（2）否（请跳至第 12 题）

10.1 如饮酒，请回答每天的饮酒量为：________________

12. 您是否有过敏性疾病或过敏药物或食物等？

（1）是　　（2）否（请跳至第 13 题）

12.1 如是，请详述：①________________

②________________

③________________

④________________

13．您目前**三个月内**是否患有以下疾病（如有，在选项上画√；如没有，则不用填写）：

中风/脑溢血/脑血栓	☐	严重冠心病/心衰	☐
急性心肌炎	☐	严重心律不齐	☐
心脏接受介入式疗法	☐	癌症	☐
癫痫	☐	抑郁症	☐
活动期消化道溃疡	☐	胃肠道功能紊乱（如慢性腹泻）	☐
甲状腺疾病	☐	脑垂体和腺垂体疾病	☐
严重肝脏疾病	☐	严重肾脏疾病	☐
III期高血压	☐	红斑狼疮/艾滋病	☐

14．您目前**三个月内**是否经常性服用以下保健品或补充剂：

亚麻子或木酚素	☐	女人馨	☐
大豆异黄酮	☐	葛根素	☐
天然元	☐		

富含异黄酮的保健品如：欣靓胶囊、沃格青春 100、金瑞芬胶囊、美雌源胶囊、春之颜胶囊、丽雌源胶囊等各种名称中直接含有异黄酮字样的保健品。

该页由研究人员填写！　　编号：________________

入选标准：以下全部选择“是”才能入选		
	是	否
（1）年龄为 50—79 周岁（1927 年 1 月 1 日至 1956 年 3 月 1 日之间出生）	☐	☐
（2）确诊的 2 型糖尿病患者	☐	☐
（3）女性阴道停止出血 1 年以上（2005 年 3 月 1 日之前停止出血）	☐	☐
（4）低密度脂蛋白≥2.9mmol/L(115mg/dl)	☐	☐
（5）生活能够自理，具有良好的记忆能力，能够自行签署知情同意书	☐	☐
**排除标准：以下全部选择“否”才能入选**		
	否	是
（1）目前或曾经（试验之前 6 个月内）使用了雌激素疗法或者固醇类激素疗法；口服或者肠道外注射用皮质固醇类药物	☐	☐
（2）任何除糖尿病以外的原因导致的高血糖症（如原发性高血糖症）	☐	☐
（3）使用胰岛素对其血糖进行控制	☐	☐
（4）试验前 3 个月内参加了其他研究项目	☐	☐
（5）急慢性感染；常规使用抗生素等会影响试验结果的药物	☐	☐
（6）癌症患者或五年内接受过放射和化学治疗者	☐	☐
（7）使用毒品或者酗酒者	☐	☐
（8）目前或曾经有过严重心脑血管疾病，可能会干扰试验的正常进行	☐	☐
（9）任何精神疾病；癫痫患者或者使用抗癫痫治疗中；使用抗抑郁药物者	☐	☐
（10）活动性消化道溃疡或者影响食物消化吸收的其他疾病	☐	☐
（11）严重甲状腺疾病（甲亢或甲退）或垂体疾病	☐	☐
（12）严重肝、肾功能疾病患者（GPT 和肌酐≥1.5 倍正常值）	☐	☐
（13）经常服用富含异黄酮或木酚素的保健品或补充剂（试验前 3 个月内）	☐	☐
（14）收缩压≥180 毫米汞柱或舒张压≥120，或III期高血压病患者	☐	☐

四、《受试者筛选表》填写说明

1　《受试者筛选表》应于筛选体检前完成，根据筛选表填写情况初步判断其是否符合入选条件，符合则参加筛选体检，不符合则无需参加筛选体检。

2　《受试者筛选表》可事先发至潜在受试者手中，由受试者填写后交至研究人员处。或者可由研究人员向潜在受试者询问填写。后一种方法较好。

3　在《受试者筛选表》上发现有问题的地方应该及时向受试者询问，以保证资料的正确性。发现不符合试验入选条件的地方，应向受试者询问清楚后决定该问题的正确性，防止潜在受试者的遗漏。

4　由研究人员填写的那一页纸可附于筛选表的最后，也可以单独打印，待研究人员收集好筛选问卷后再行填写。

5　在相应的□内填写数字或用符号“√”表示选择，数字不要填出编码格外。

6　《受试者筛选表》应妥善保存，所有信息应及时输入电脑数据库中。所有受试者筛选结束后，《受试者筛选表》等受试者信息资料及时转交中科院营养所以进行受试者分组。

五、筛选体检表

姓名：	编号：
性别：	体检日期:2006 年___月___日
检查员:	质控员:

亚麻子健康改善项目

（筛 选 用 体 检 表）

1. 空腹：□是 □否

2. 抽血：□完成 □未完成

3. 身高：□□□.□厘米

（精确到 0.1 厘米）

4. 体重：□□□.□公斤

衣服重量： □.□公斤

实际重量：□□□.□公斤

（精确到 0.1 公斤）

5. 腰围:□□□.□厘米

（精确到 0.1 厘米）

6. 臀围:□□□.□厘米

（精确到 0.1 厘米）

7. 血压和脉搏：

第一次测量：

收缩压□□□毫米汞柱

舒张压□□□毫米汞柱

脉 搏□□□/分钟

第二次测量：

收缩压□□□毫米汞柱

舒张压□□□毫米汞柱

脉 搏□□□/分钟

第三次测量：

收缩压□□□毫米汞柱

舒张压□□□毫米汞柱

脉 搏□□□/分钟

第五部分、病例报告表

一、病例报告表

内部资料，限相关人员使用，请勿外传

编号：____________________

亚麻子健康改善项目

病 例 报 告 表

上海市科学技术委员会项目

中国科学院上海生命科学研究院营养科学研究所编制

2006 年 1 月

研究机构名：	中国科学院上海生命科学研究院营养科学研究所

胶囊编号：　　□□□

受试者姓名首字母缩写（大写）　　姓□□名□□

*如果姓氏缩写只有一个字母，请填写在后面一格内

知情同意书签署	日期：2006 年□□月□□日

研究者签名	
日期：	2006 年□□月□□日

最终确认	本病例报告表中的内容以及修改内容已经确认<br>主要研究者（签名）<br>最终确认日：　2006 年□□月□□日

初访：背景资料

受试者背景资料

姓名：姓☐☐名☐☐（首字母缩写）			
性别：☐1.男 ☐2.女		出生日期：19☐☐年☐☐月☐☐日	
民族：		婚姻状况：	
文化程度：		就业状况：	
糖尿病诊断时间：		高血脂诊断时间：	
过敏物或药物			

第一阶段面对面随访一

随访日期：2006年☐☐月☐☐日		询问者签名：	
随访地点：		访问时间：☐1、空腹 ☐2、饭后 ☐.☐小时	
询问项目	询问结果		
服用胶囊情况	服用数量 ☐☐☐颗	回收数量	☐☐☐颗
胶囊发放	☐瓶，共计☐☐☐颗	药物使用*	☐改变 ☐未改变
不良事件*	☐次 编号	☐☐/☐☐/☐☐	
身体状况			
受试者主诉			
采取措施			

*若有药物改变，请填写《合并用药表》；若有不良事件，请填写《不良事件表》。

第一阶段结束时询问结果

询问项目	询问结果			
日期： 2006 年☐☐月☐☐日			询问者签名：	
服用胶囊情况	服用数量	☐☐☐颗	回收数量	☐☐☐颗
不良事件	☐次	编号	☐☐/☐☐/☐☐	
药物服用	☐改变 ☐未改变		若有药物改变，请填写《合并用药表》；若有不良事件，请填写《不良事件表》。	
身体状况				
受试者主诉				
采取措施				

第一阶段电话随访一：服用胶囊情况和不良事件

随访日期：2006 年☐☐月☐☐日			询问者签名：
询问项目	询问结果		备注
服用胶囊情况	是否按时按量服用胶囊	☐是 ☐否	
不良事件	☐次	编号	☐☐/☐☐/☐☐
药物服用	☐改变 ☐未改变		若有药物改变，请填写《合并用药表》；若有不良事件，请填写《不良事件表》。
身体状况			
受试者主诉			
采取措施			

休息阶段电话随访一

随访日期：2006 年□ □月□ □日		询问者签名：	
询问项目	询问结果		
不良事件*	□次	编号	□ □/□ □/□ □/□ □
药物服用*			
身体状况			
受试者主诉			
采取措施			

*若有药物改变，请填写《合并用药表》；若有不良事件，请填写《不良事件表》。

休息阶段电话随访二

随访日期：2006 年□ □月□ □日		询问者签名：	
询问项目	询问结果		
不良事件*	□次	编号	□ □/□ □/□ □/□ □
药物服用*			
身体状况			
受试者主诉			
采取措施			

*若有药物改变，请填写《合并用药表》；若有不良事件，请填写《不良事件表》。

不良事件记录表

编号	1	2	3
不良事件			
起始日期	2006 年<br>☐☐月☐☐日	2006 年<br>☐☐月☐☐日	2006 年<br>☐☐月☐☐日
终止日期	2006 年<br>☐☐月☐☐日	2006 年<br>☐☐月☐☐日	2006 年<br>☐☐月☐☐日
严重程度	☐1 轻度<br>☐2 中度<br>☐3 重度	☐1 轻度<br>☐2 中度<br>☐3 重度	☐1 轻度<br>☐2 中度<br>☐3 重度
是否 SAE	☐1 是<br>☐2 否	☐1 是<br>☐2 否	☐1 是<br>☐2 否
治疗	☐1 无<br>☐2 药物<br>☐3 处理<br>☐4 其他 ______	☐1 无<br>☐2 药物<br>☐3 处理<br>☐4 其他______	☐1 无<br>☐2 药物<br>☐3 处理<br>☐4 其他______
因果关系	☐1 无关<br>☐2 可能无关<br>☐3 可能有关<br>☐4 很可能有关<br>☐5 肯定有关	☐1 无关<br>☐2 可能无关<br>☐3 可能有关<br>☐4 很可能有关<br>☐5 肯定有关	☐1 无关<br>☐2 可能无关<br>☐3 可能有关<br>☐4 很可能有关<br>☐5 肯定有关
采取措施	☐1 无<br>☐2 中断试验胶囊<br>☐3 终止试验胶囊<br>☐4 其他 ______	☐1 无<br>☐2 中断试验胶囊<br>☐3 终止试验胶囊<br>☐4 其他______	☐1 无<br>☐2 中断试验胶囊<br>☐3 终止试验胶囊<br>☐4 其他 ______
转归	☐1 恢复<br>☐2 恢复后但有后遗症<br>☐3 改善<br>☐4 继续<br>☐5 恶化<br>☐6 致死<br>☐7 不明	☐1 恢复<br>☐2 恢复后但有后遗症<br>☐3 改善<br>☐4 继续<br>☐5 恶化<br>☐6 致死<br>☐7 不明	☐1 恢复<br>☐2 恢复后但有后遗症<br>☐3 改善<br>☐4 继续<br>☐5 恶化<br>☐6 致死<br>☐7 不明

严重不良事件报告表（SAE）

<table>
<tr><td colspan="2">报告类型</td><td colspan="3">☐首次报告 ☐随访报告 ☐总结报告</td><td colspan="2">报告时间： 年 月 日</td></tr>
<tr><td colspan="2">医疗机构及专业名称</td><td colspan="3"></td><td colspan="2">电话</td></tr>
<tr><td colspan="2">申报单位名称</td><td colspan="3"></td><td colspan="2">电话</td></tr>
<tr><td colspan="2" rowspan="2">试验用药品名称</td><td colspan="5">中文名称：</td></tr>
<tr><td colspan="5">英文名称：</td></tr>
<tr><td colspan="2">药品注册分类及剂型</td><td colspan="5">分类：☐中药 ☐化学药 ☐治疗用生物制品 ☐预防用生物制品☐其它______
注册分类：_______________ 剂型:__________________</td></tr>
<tr><td colspan="2">临床研究分类</td><td colspan="3">☐ I 期 ☐ II 期 ☐III 期 ☐IV期
☐生物等效性试验 ☐临床验证</td><td colspan="2">临床试验适应症：</td></tr>
<tr><td rowspan="2">受试者基本情况</td><td>姓名拼音缩写:</td><td>出生日期:</td><td>性别: ☐男 ☐女</td><td>身高(cm)：</td><td colspan="2">体重(Kg)：</td></tr>
<tr><td colspan="6">合并疾病及治疗：☐有 ☐无
1．疾病：__________ 治疗药物：__________ 用法用量：_______________
2．疾病：__________ 治疗药物：__________ 用法用量：_______________
3．疾病：__________ 治疗药物：__________ 用法用量：_______________</td></tr>
<tr><td colspan="2">SAE 的医学术语(诊断)</td><td colspan="5"></td></tr>
<tr><td colspan="2">SAE 情况</td><td colspan="5">☐ 死亡 ______年___月___日
☐ 导致住院 ☐延长住院时间 ☐伤残 ☐功能障碍
☐ 导致先天畸形 ☐危及生命 ☐其它______________</td></tr>
<tr><td colspan="3">SAE 发生时间： _______年___月___日</td><td colspan="4">研究者获知 SAE 时间： _______年___月___日</td></tr>
<tr><td colspan="2">对试验用药采取的措施</td><td colspan="5">☐继续用药 ☐减小剂量 ☐药物暂停后又恢复 ☐停用药物</td></tr>
<tr><td colspan="2">SAE 转归</td><td colspan="5">☐ 症状消失（后遗症 ☐有 ☐无） ☐症状持续</td></tr>
<tr><td colspan="2">SAE 与试验药的关系</td><td colspan="5">☐肯定有关 ☐可能有关 ☐可能无关 ☐肯定无关 ☐无法判定</td></tr>
<tr><td colspan="2">SAE 报道情况</td><td colspan="5">国内： ☐有 ☐无 ☐不详； 国外： ☐有 ☐无 ☐不详</td></tr>
</table>

SAE 发生及处理的详细情况：

报告单位名称： 报告人职务/职称： 报告人签名：

合并用药表

	伴随用药（请填写通用名）				用药目的	开始日	终止日
	名称	剂量	频率	给药途径			
1				☐ 1 口服<br>☐ 2 肌内注射<br>☐ 3 静脉点滴<br>☐ 4 静脉推注<br>☐ 5 其他<br>( )	☐ 1 治疗自身疾病<br>☐ 2 治疗不良事件<br>(编号)<br>☐ 3 其他<br>( )	2006 年<br>☐☐月<br>☐☐日	2006 年<br>☐☐月<br>☐☐日
2				☐ 1 口服<br>☐ 2 肌内注射<br>☐ 3 静脉点滴<br>☐ 4 静脉推注<br>☐ 5 其他<br>( )	☐ 1 治疗自身疾病<br>☐ 2 治疗不良事件<br>(编号)<br>☐ 3 其他<br>( )	2006 年<br>☐☐月<br>☐☐日	2006 年<br>☐☐月<br>☐☐日
3				☐ 1 口服<br>☐ 2 肌内注射<br>☐ 3 静脉点滴<br>☐ 4 静脉推注<br>☐ 5 其他<br>( )	☐ 1 治疗自身疾病<br>☐ 2 治疗不良事件<br>(编号)<br>☐ 3 其他<br>( )	2006 年<br>☐☐月<br>☐☐日	2006 年<br>☐☐月<br>☐☐日
4				☐ 1 口服<br>☐ 2 肌内注射<br>☐ 3 静脉点滴<br>☐ 4 静脉推注<br>☐ 5 其他<br>( )	☐ 1 治疗自身疾病<br>☐ 2 治疗不良事件<br>(编号)<br>☐ 3 其他<br>( )	2006 年<br>☐☐月<br>☐☐日	2006 年<br>☐☐月<br>☐☐日
5				☐ 1 口服<br>☐ 2 肌内注射<br>☐ 3 静脉点滴<br>☐ 4 静脉推注<br>☐ 5 其他<br>( )	☐ 1 治疗自身疾病<br>☐ 2 治疗不良事件<br>(编号)<br>☐ 3 其他<br>( )	2006 年<br>☐☐月<br>☐☐日	2006 年<br>☐☐月<br>☐☐日

完成、退出及提前终止试验表

<table>
<tr><td>试验结束日期</td><td colspan="2">2006 年□□月□□日</td></tr>
<tr><td>最后一次服用试验胶囊日期</td><td colspan="2">2006 年□□月□□日</td></tr>
<tr><td>参加试验日期</td><td colspan="2">2006 年 月 日—2006 年 月 日</td></tr>
<tr><td>总共服用胶囊数目</td><td colspan="2">□□□颗</td></tr>
<tr><td>是否有在试验结束前破盲的情况</td><td colspan="2">□1 是 □2 否
如是，请注明理由：</td></tr>
<tr><td colspan="3">如果是退出及提前终止试验的病例，请填写下表</td></tr>
<tr><td>剔除理由（选择一个最主要的理由，请勿多选）</td><td colspan="2">□1 发生急性心肌梗死
□2 严重不良事件（编号： ）
□3 发生其他可能影响研究结果的疾病
□4 服用了本研究禁止的药物
□5 主要研究者认为有其他理由终止
请说明：____________________</td></tr>
<tr><td>退出理由</td><td colspan="2">□ 受试者要求退出
□ 1 受试者失访 ____________________
□ 2 其他理由 ____________________</td></tr>
<tr><td>请描述退出的原因和过程</td><td colspan="2"></td></tr>
<tr><td rowspan="3">追踪</td><td>追踪日</td><td>200 年 月 日</td></tr>
<tr><td>追踪方法</td><td>□1 信件 □2 电话
□3 其他方法( )</td></tr>
<tr><td>追踪结果</td><td>□1 健在
□2 无法确认
□3 其他( )</td></tr>
<tr><td>备注</td><td colspan="2"></td></tr>
</table>

二、《病例报告表》填写说明

（一）、总体要求

1 病例报告表的填写，必须使用钢笔或圆珠笔，字迹要端正清楚，不得潦草模糊、随意涂改，阿拉伯数字必须按正楷体书写，不得用自由体。

2 在相应的□内填写数字或用符号“√”表示选择，数字和编码不要填出编码格外。表中项目所列情况如没有则填写“无”，如不清楚则填写“不清楚”。如有疑问，请询问受试者，保证数据正确。

3 用文字或数字填写的文字填在指定的横线上。数字一律由右至左按个十百千万填写，左边格子不满用“0”补足。数字答案按指定单位的整数或小数位数填，小数后面的“0”不能省略填写。如体重 59kg，正确填法为

0	5	9	.	0

4 除有专项说明外，逻辑上不用调查的不填任何符号。

5 调查表填错后的更正方法：先在错误项的文字或数字上用双横线划去，使修改前的内容仍清晰可见，在划线上方另行填写正确文字或数字，切勿在原数码上涂改；如有必要，在修改处注明修改日期并签名。不得使用涂改液或涂改带。例如，将 2 改成 4；

4 XXX, XX 年 XX 月 XX 日

正确的改法为： ~~2~~

6 病例报告表中的数据必须与原始数据一致。如有出入，须注明理由并签字。

7 试验结束后，研究员在对病例报告表的填写和修改确认以后，移交病例报告表至监查员或数据管理部门。如需修改，将需修改的内容填入数据疑问表提交至监查员。

（二）、具体内容

1 在首页准确粘贴受试者编号。次页准确填写胶囊编号，受试者姓名首字母大写等信息。注意：最终确认必须是在整个试验结束后对该病例报告表确认完全后填写。

2 根据受试者筛选问卷或基线一般调查问卷以及询问结果填写受试者背景资料。

3 本病例报告表将面对面随访和电话随访分开，将两个阶段的资料分开。按照试验方案，受试者被要求每三周前往华东医院面对面随访一次，研究人员至少对受试者每周电话随访一次。因此，面对面随访和电话随访内容不同，分开填写。

4 面对面随访：按照要求填写相关信息。“身体状况”是研究人员根据受试者主诉及面对面观察填写其综合身体状况。填写时只需要填写五个等级即可：很差，较差，一般，较好，很好。同

时，此栏中还需要询问受试者的饮食和运动情况是否有改变，如有显著改变，则需要填写改变的情况，如没有改变或改变很少，只需要填写 “保持原有生活方式”。“受试者主诉”主要填写受试者服用胶囊后的身体反应或变化。“采取措施”主要填写根据其身体状况及不良事件情况对其是否能参与试验做出判断，如继续试验、暂停胶囊、终止试验等等。胶囊的发放和回收按照实际情况填写，回收数量的计算由营养所专人负责，每次面对面随访完成后及时反馈给随访人员填写。“药物使用”若有改变，则需要填写《合并用药表》。若有不良事件，则需要填写《不良事件表》。

5 电话随访：每周至少对每位受试者电话随访一次。按要求填写相关内容。“备注”中填写未按时按量服用胶囊的原因。“药物使用”、“不良事件”、“身体状况”、“受试者主诉”和“采取措施”按照第 5 条的要求填写。

6 由于第二阶段的面对面随访和电话随访的内容和第一阶段的一样，所以此操作手册中只列举了第一阶段的表格形式，第二阶段的填写要求是一样的。

7 合并用药表格的填写：合并用药是指除服用试验用胶囊以外服用的任何药物。“剂量”需要填写每次服用时的颗数或者重量，“频率”需要填写每天服用的次数。若一种药物常规或经常使用，只需要填写在一行中，写明服药起始和结束日期。若改变剂量，则需另起一行填写。

8 完成或退出试验时填写《完成、退出及提前终止试验表》，按照要求和内容填写即可。

9 不良事件表的填写说明：

【严重程度分类】

轻度：对受试者日常生活无影响。

中度：影响受试者日常生活。

重度：限制了受试者日常生活，需要治疗或中止本试验。

【严重不良事件的定义】

如果选择是严重不良事件，务必填写《严重不良事件报告表》。

严重不良事件与事件严重程度的“严重”不是同一个概念。严重不良事件是指：①死亡；②危及生命；③须住院治疗或延长原来的住院时间，或导致入院；④严重或永久的残疾或失能；⑤严重的医疗事件，指不一定马上危及生命、导致死亡或住院的情况，但是极大的危害了受试者，需要采取措施预防以上严重后果的发生。

【因果关系判断】

是指该不良事件是否由于应用试验胶囊所引起。如果判断是由试验胶囊所引起的不良事件称为不良反应。

因果关系判断标准：

(1) 无关：该不良事件明确与试验用胶囊无关。

(2) 可能无关：该不良事件与试验用胶囊可能无关。

(3) 可能有关：该不良事件与试验用胶囊可能有关。

(4) 很可能有关：该不良事件与试验用胶囊有关的可能性较大。

(5) 肯定有关：该不良事件与试验用胶囊的关系很明确。

一般都以后 3 种判为与胶囊有关。

具体的判断需要由双方主要研究人员组成的专家委员会决定。随访的研究人员在接到不良事件报告后应及时通知主要研究人员，不应擅自作出关系判断。

判断方法：

关系	肯定有关	很可能有关	可能有关	可能无关	肯定无关
有合理的时间顺序	+	+	+	+	-
已知的胶囊反应类型	+	+	+	-	-
停药后反应减轻或消失	+	+	±	±	-
再次给药后反应反复出现	+	?	?	?	-
无法用受试者疾病来解释	+	+	-	±	-

第六部分、问卷调查

一、基线一般调查问卷

编号（CODE）________________

调查日期 2006年 月 日

调查员 ______/______

质控员 ______/______

亚麻子健康改善计划

（基线一般调查问卷）

姓名：________________

住址：________________

电话：________________

上海市科学技术委员会项目

中国科学院上海生命科学研究院营养科学研究所编制

2006年1月

人口学资料

1.性别：(1)男　　　　(2)女 □

2.出生日期：19□□年□□月□□日

3.目前全家共同生活人口数为：□□人

4.民族？ □

（1）汉族　（2）回族　（3）其他

5.婚姻状况？ □

（1）已婚/同居　（2）从未结婚　（3）离异/分居　（4）丧偶

6.受学校教育时间共有：□□年

文化程度？ □

（1）没有上过学　（2）小学　（3）初中　（4）高中、中等职业教育
（5）大专、本科　（6）研究生

7.职业（如果目前领社会保障金、退休或待业，则指最后从事过的职业）？ □

（1）工人、农民等从事重体力活动的职业
（2）普通职员、个体工商、商业服务人员等从事中等体力活动的职业
（3）高级管理人员、行政管理人员、专业技术人员等从事轻体力活动的职业
（4）军人
（5）家庭主妇或持家
（6）其他，具体为______________________

8.目前的就业状态? □

（1）正常工作　（2）返聘　（3）退休　（4）待业　（5）领低保

9.若已退休，则已退休了□□年□□月

10.去年全家总收入共计多少元？ □

（1）低于 5，000 元　（2）5，000－9，999 元　（3）10，000－19，999 元
（4）20，000－29，999 元　（5）30，000－39，999 元　（6）40，000－59，999 元
（7）60，000－79，999 元　（8）80，000 元以上　（9）不清楚

11.去年您个人总收入共计多少元？（包括子女给的赡养费） □

（1）低于 2，000 元　（2）2，000－4，999 元　（3）5，000－9，999 元
（4）10，000－19，999 元　（5）20，000－39，999 元　（6）40，000－59，999 元
（7）60，000 元以上　（8）不清楚

关于您的糖尿病

12.您的亲属是否曾被诊断患有下列疾病？（可多选，1＝是，2＝否，3=不清楚）

A,糖尿病	父母	☐
	兄弟姐妹	☐
	如是，是否有人在 40 岁以前？	☐
B,冠心病/心肌梗塞	父母	☐
	兄弟姐妹	☐
	如是，是否有人在 50 岁以前？	☐
C,中风	父母	☐
	兄弟姐妹	☐
	如是，是否有人在 60 岁以前？	☐
D,高血压病	父母	☐
	兄弟姐妹	☐

13.过去一个月内，您是否被下列症状困扰？如果有，程度如何？（填写相应的数字到方框中）

	从没有	偶尔有	经常有	
A,感到头晕或头重脚轻	1	2	3	☐
B,皮肤瘙痒	1	2	3	☐
C,突然出现乏力，头昏，心跳加快，出虚汗，发抖，进食后可以缓解（发生低血糖）	1	2	3	☐
D,视力模糊（戴眼镜不能纠正）	1	2	3	☐
E,足部的针刺感，疼痛感，麻木感，或烧灼样感觉	1	2	3	☐
F,脚趾或足部的溃疡或感染	1	2	3	☐
G,多尿	1	2	3	☐
H,口干或周期性的极度口渴	1	2	3	☐

14．过去一年中，您服用过治疗糖尿病的西药吗？ ☐

（1）是　　　　（2）否 →转到 15 题　　　（3）不清楚 →转到 15 题

14.1 您是否按医嘱服用药物？ ☐

（1）从未　（2）少数时间　(3)有一些时间　（4）大多数时间　（5）全部时间

14.2 这些药物对减轻您糖尿病症状的效果如何？ ☐

（1）完全无效（2）有一点效果 (3)效果一般 （4）有较大效果（5）非常有效

15．在过去一年中，您采用过糖尿病辅助治疗吗？ ☐

（1）是　　　　（2）否 →转到 16 题　　　（3）不清楚 →转到 16 题

15.1 您采用过以下的哪种辅助治疗方式？

	是	否	
A, 调整饮食	1	2	☐
B, 运动	1	2	☐
C, 中草药治疗	1	2	☐

D,其他，具体为____________________

15.2 以上辅助治疗对减轻您糖尿病症状的效果如何？ ☐

（1）完全无效（2）有一点效果 (3)效果一般 （4）有较大效果（5）非常有效

16.下列检测您多久做一次？

	从不检测	每年 1 次	每年 2 次或以上	不清楚	
A,糖化血红蛋白	1	2	3	4	☐
B,血脂	1	2	3	4	☐
C,肾脏功能检查	1	2	3	4	☐
D,眼科并发症检查	1	2	3	4	☐
E,足部并发症检查	1	2	3	4	☐

17．您多久检测一次血糖？ ☐

（1）每天 1 次或以上（2）每周 1-6 次 (3)每月 1-2 次（4）偶尔（5）从不检测 →转到 18 题

17.1 您是否自己拥有血糖仪？ ☐

（1）是　　　　（2）否

17.2 您通常在那里检测血糖？ （单选题） □

（1）家里 （2）社区医务室 （3）医院 (4)其他，具体为________

17.3 您通常在什么时间检测血糖？ （单选题） □

（1）早晨空腹（2）餐后 (3)不定时 （4）其他，具体为________

18.您对下列糖尿病知识的了解程度：

	不了解	了解较少	一般	了解较多	非常了解	
A,饮食治疗和食物选择	1	2	3	4	5	□
B,体力活动和锻炼	1	2	3	4	5	□
C,降糖药的使用	1	2	3	4	5	□
D,高血糖/低血糖的处理	1	2	3	4	5	□
E,饮酒/吸烟和糖尿病	1	2	3	4	5	□

19.您对下列糖尿病控制的看法：

	坚决不同意	不同意	不确定	同意	非常同意	
A,血糖是可以控制在良好水平的	1	2	3	4	5	□
B,体重能够控制好的	1	2	3	4	5	□
C,饮食控制、服药、锻炼等有助于糖尿病的控制	1	2	3	4	5	□
D,情绪的控制对糖尿病也很有利	1	2	3	4	5	□

吸烟

20.目前是否吸烟？ □

（1）是 （2）否（转到第 21 问）

20.1 每天的吸烟量是：(本题回答后转到 24 题) □

(1)偶尔吸 （2）1－10 支 （3）10－20 支 （4）20 支以上

21.过去是否吸烟？ □

（1）是 （2）否（转到第 23 问）

22.戒烟时年龄： □□周岁

23.目前在工作、家庭生活环境中是否被动吸烟？ □

（1）是 （2）否

饮酒

24.您是否喝酒？

种类	从不	偶尔	每天少于1杯	每天1-2杯以内	每天2-4杯	
A,啤酒（200毫升/杯）	1	2	3	4	5	□
B,红酒（200毫升/杯）	1	2	3	4	5	□
C,黄酒（200毫升/杯）	1	2	3	4	5	□
D,白酒（50毫升/杯）	1	2	3	4	5	□

注：瓶装啤酒一般有350毫升和640毫升两种，罐装啤酒一般为330毫升；
红酒200毫升为4两；黄酒200毫升为3两；白酒50毫升为1两。

健康状况

心理健康

25.过去一个月内，您有多少时间处于以下状态？请选择一个最接近的答案。

	从来没有	少数时间	一些时间	多数时间	大多数时间	全部时间	
A,自我感觉心态平静	1	2	3	4	5	6	□
B,自我感觉精力充沛	1	2	3	4	5	6	□
C,情绪低落、什么事也不能令您快乐	1	2	3	4	5	6	□
D,感觉到很疲倦	1	2	3	4	5	6	□

自我健康评价

26.您认为自己的健康状况如何？ □

（1）非常好 （2）很好 （3）一般 （4）比较差 （5）非常差 （6）不清楚

27.和一年前这个时候相比，您认为目前的身体健康状况如何？ □

（1）好很多 （2）好一些 （3）差不多 （4）差一些 （5）差很多 （6）不清楚

28.同一年前相比，您的体重是: □

（1）增加 （2）基本保持不变 （3）下降 （4）不清楚

29.在近一年内您曾试图改变体重吗？ □

（1）减重 （2）增重 （3）未尝试改变

睡眠质量

30.在过去一个月，您的睡眠好吗？ □

（1）好 （2）一般 （3）不好 （4）说不清

31.在过去一个月，您是否有难以入睡的情况（包括半夜醒来后）？ □

（1）经常有 （2）偶尔有 （3）没有 （4）说不清

32.在过去一个月，您平均每天睡眠时间有多长（包括午睡）？

□□小时□□分钟

医疗保健

33.您目前享受何种医疗保险？（可多选，1=是，2=否，3＝不清楚）

A,社会医疗保险	□	B,商业医疗保险	□
C,公费医疗（100%报销）	□	D,其它医疗保险	□

34.过去一年您个人医药费的总支出是： □

（1）支出□□□□□元 （2）记不清

35．是否患有以下疾病（可多选，1=是，2=否，3=不清楚）

A,风湿性/类风湿性关节炎	□	B,关节炎	□
C,骨折	□	D,骨质疏松	□
E,骨质增生	□	F,青光眼/白内障	□
G,脂肪肝	□	H, 胆囊疾病	□
I,肾脏疾病	□	J,胃、肠、胰腺等消化系统疾病	□
K,肺部疾病	□	L,心脑血管疾病	□
M,皮肤瘙痒或其他慢性皮肤病	□	N,前列腺疾病（男性）	□
O,子宫疾病（女性）	□	P,卵巢疾病（女性）	□

36．女性请回答：您是否接受过卵巢（单侧或双侧）全切除术？ □

（1）是 （2）否

36.1 如是，请回答进行手术的时间：□□年□□月 或当时年龄：□□周岁

体力活动

37．请回忆在**过去一个月内**平均**每周**所做的以下体力活动的总时间：

活动名称	无	1 小时以内	1-2 小时以内	2-4 小时以内	4-10 小时以内	10 小时或以上	
A,跑步、游泳	1	2	3	4	5	6	□
B,搬运重物	1	2	3	4	5	6	□
C,其他重体力活动	1	2	3	4	5	6	□
D,骑自行车	1	2	3	4	5	6	□
E,乒乓球、羽毛球等	1	2	3	4	5	6	□
F,照看孩子、老人	1	2	3	4	5	6	□
G,做操打拳、跳舞	1	2	3	4	5	6	□
H,其他中等强度体力活动	1	2	3	4	5	6	□
I,一般家务劳动	1	2	3	4	5	6	□
J,养花种草	1	2	3	4	5	6	□
K,其他轻体力活动	1	2	3	4	5	6	□
L,步行	1	2	3	4	5	6	□
M,散步、遛狗等	1	2	3	4	5	6	□
N,在家中走动时间	1	2	3	4	5	6	□
O,工作或乘车时坐立	1	2	3	4	5	6	□
P,在家中坐立（如看电视、吃饭、看书、编织、下棋等）	1	2	3	4	5	6	□

重体力活动是指需要花费大力气完成，呼吸较平常明显增强的活动。

中等强度体力活动是指需要花费中等力气完成，呼吸较平常稍微增强的活动。

轻体力活动是指仅需花费少量力气完成的工作。

这里的**步行**包括你工作时的行走，上下班、买菜、购物以及为了锻炼身体进行的步行。

药物使用登记：

编号：__________ 姓名：___________ 日期：2006 年____月___日 调查员：______________

1.请登记**目前**服用用药物情况（常规服用以及一周内服用过的药物）：

药物种类	药物名称	开始使用时间	剂量（颗/天）
A，降糖药	1、		
	2、		
	3、		
B，降脂药	1、		
	2、		
	3、		
C，降压药	1、		
	2、		
	3、		
D，其他	1、		
	2、		
	3、		
	4、		
	5、		

2.请登记最近**目前**服用的保健品和营养补充剂情况：

保健品或营养补充剂名称	开始使用时间	剂量

膳食信息

1．为了解您的饮食营养是否合理，请回忆您**过去一年中**以下食物的食用次数和数量。

（填写说明：在次数前打 √，并填写食用数量（**指生食，净重**））

豆类及其制品	进 食 次 数						平均每次食用量	食物编码(由科研人员写)
	每日 2 次或以上	每日 1 次	隔日 1 次	每周 1-2 次	每月 1-2 次	很少或不吃	两	
A，黄豆、黑豆、大豆								
B，豆腐								
C，豆浆								
D，黄豆粉								
E，豆芽(绿、黄豆芽)								
F，豆腐花(豆腐脑)								
G，其他豆制品（豆腐干、百页、素鸡、腐竹、豆腐皮、油豆腐、豆奶粉等）								
H，其他豆类（蚕豆、扁豆、毛豆、豇豆、绿豆、赤豆、芸豆等）								
I，酱类（豆豉/黄酱/豆瓣酱、腐乳等）								

其他	进 食 次 数						平均每次食用量	食物编码(由科研人员写)
	每日 2 次或以上	每日 1 次	隔日 1 次	每周 1-2 次	每月 1-2 次	很少或不吃	两	
I，杂粮(小米、玉米、燕麦、荞麦、高粱等)								
J，坚果（花生/瓜子/核桃/南瓜子等）								
K，芝麻								
L，红茶、绿茶								
M，水果类								

2. 以家庭为单位按月询问，全家一起就餐 ____ 人

食用油	全家食用量（斤/月）	不吃填 0	食物编码（由科研人员填写）
A，植物油（请注明：　　　　　）			
B，猪油，黄油			
C，沙拉酱，沙拉油			
D，其它食用油(请注明：　　　　)			

二、《基线一般调查问卷》填写说明

（一）、总体要求

1 调查表的填写，必须使用钢笔或圆珠笔，字迹要端正清楚，不得潦草模糊、随意涂改，阿拉伯数字必须按正楷体书写，不得用自由体。

2 将选中的选项的编码，填在调查表右侧相应的格子内，数字和编码不要填出编码格外。

3 用文字或数字填写的文字填在指定的横线上。数字一律由右至左按个十百千万填写，左边格子不满用“0”补足。数字答案按指定单位的整数或小数位数填，小数后面的“0”不能省略填写。如体重 59kg，正确填法为

0	5	9	.	0

4 除有专项说明外，逻辑上不用调查的不填任何符号。

5 调查表填错后的更正方法：先在错误项的文字或数字上用双横线划去，在划线上方另行填写正确文字或数字，切勿在原数码上涂改；如有必要，需写上修改人的姓名。例如，将 2 改成 4；

4 XXX, XX 年 XX 月 XX 日

正确的改法为： ~~2~~

6 开始问卷前在首页准确填写调查日期（当天）和调查员姓名（两名）；正楷字填写被调查者姓名、住址和联系电话（应为家庭/单位电话，如有手机可加填手机号，若无家庭/单位电话填居委会）。

7 对于有可供填写不清楚或者记不清的选项，调查员应该根据实际情况判断，不能直接读出来让受试者选择。

8 问卷的调查者应尽量使用贴近生活以及老年人实际情况的语言进行提问，避免书面化。可以使用上海话提问。

（二）、问题部分

只针对需解释问题，按问题顺序号排列

<u>人口学资料</u>

2 出生日期：按户籍册或身份证填写，必须核对其身份证后填写。

3 目前全家共同生活人口数为共同用餐的人数总和，本题目的为调查人均年收入，如晚上共同用餐后离去也应包括在内。

5 婚姻状况中同居指在一起生活但未办理结婚手续。

6 文化程度中研究生指硕士及以上学历。受教育时间应包括全部受教育年限，如某人本科学历，

但大学学历为通过高教自学考试获得，历时5年完成，则受学校教育时间应为：小学（6年）＋初中（3年）＋高中（3年）＋高自考（5年）＝17年。

私塾按上学年限计算，上过6年以内的为小学文化程度，9年以内的为初中文化程度，依此类推。另外，高小归类为小学程度。

7 农民指主要从事农业劳动的个人，生活在农村但主要在乡镇企业工作的个人如电工、生产线操作等应为工人。工人中除表上标注外，还应包括所有蓝领阶层如司机、清洁工等。商业、服务人员指商业、服务业从业人员如售货员、服务员、厨师、理发师等；虽在饭店工作，但工作是清洁工应算工人。高级管理人员包括外企、国企、民营企业、私企等中上层管理人员；行政管理人员指居委会/村委会成员及以上各级政府机关行政人员。专业技术人员包括教师、医生、科研工作者等从事专业技术和脑力活动的人员；从来无业按持家归类。如仍没有发现符合以上条件者，可选择“6 其他”，并可在旁边附注其职业。

8 退休或待业同时领低保选填退休或待业。

9 如没有退休，此题无需提问和填写。如退休，可先问哪一年哪一月退休，或退休时年龄，在计算一共到现在的退休时间。

11 总收入指过去一年全部收入，也应包括子女供给的收入。

关于您的糖尿病

12 此题主要考察受试者的家族遗传背景。如父母和兄弟姐妹没有相应的疾病，即填写数字为3或2时，无需询问第三问“如是，是否有人在40/50/60岁以前？”这个问题，也不需要在上面填写任何数字。

13 此题主要考察由糖尿病导致的一些并发症症状，根据受试者回答如实填写。可先问受试者有没有过这些症状，再问其频率！注意提醒受试者此处需要回答的是“**过去一个月**”的症状！

14 注意此处为“**过去一年**”的服用糖尿病药物的情况！此处填写的为**西药**服用情况，服用中药等算作辅助疗法。

15 此处仍为“**过去一年**”！若受试者不知道辅助治疗的定义，可按15.1的次序依次询问。消渴丸为中药，算作辅助疗法；服用保健品算作辅助疗法的“D,其他，具体为______”，并在横线处填写保健品名称；多吃苦荞麦、杂粮等算作调整饮食类。

17 此题中17.2和17.3为单选题，请填写“**通常**”检测血糖的地点和时间。

18和19 此二题根据受试者自己对于糖尿病相关知识和控制情况的了解程度进行填写。

吸烟

20 目前是否吸烟?

指吸烟 6 个月及以上。

21 过去是否吸烟?

指过去吸烟至少 6 个月，现在戒烟 6 个月及以上。

23 指工作、家庭生活环境中有否被动吸烟，逛商场、外出用餐等被动吸烟不算。提问时不用说“被动吸烟”，而可问“工作地点或家中有没有人抽烟，是否影响到你？”

<u>饮酒</u>

24 请看清每杯容量：啤酒、红酒、黄酒为 200 毫升/杯，白酒为 50 毫升/杯。可根据注释进行“毫升”和“两”之间的换算。

<u>心理健康</u>

25 这是一组评价老年人自我心理感受（抑郁）的问题。根据产生该种感受的频度填写相应选择。

<u>自我健康评价</u>

26 和 27 为受访者自我评价，是反映受访者的主观感受，据实记录受访者的回答。

29 用过节食、锻炼、服减肥药等方法进行减重；或由于医生建议节食为被动减重。

<u>睡眠质量</u>

31 在过去一个月，你是否有难以入睡的情况（包括半夜醒来后）?

经常指有规律且每周 3 次及以上；偶尔指无规律且每周 2 次及以下。

32 指一天 24 小时内的睡眠时间。请具体填写，并精确到分钟。具体睡眠时间的计算通常是指从晚上开始入睡（而非上床）到早上醒来后整个熟睡的时间，并加上中午午休时间的总和。如因睡眠不佳，一夜中时醒时睡，则计算各次累计的入睡时间总和。如因上夜班，通常在白天休息，则用相同方法计算白天睡眠的时间。具体询问时，还请特别说明，所了解的是在通常情况下的平均睡眠时间，应排除在某些特殊情况下（如偶尔晚上加班加点等）睡眠时间的波动的影响。如果回答如“7、8 个小时吧!”则填写中间数字“7 小时 30 分钟”。

<u>医疗保健</u>

33 公费医疗指医药费百分之百报销。

34 医药费总支出指报销前的花费，有的人可能花费 1600 元报销 1000 元，但医药费总支出应填 1600 元。右边的空格中必须填写数字。

35　只填目前是否有即可。骨折则填写是否有过。前列腺疾病女性不问、不填。子宫疾病和卵巢疾病男性不问、不填。

女性回答问题部分

36　部分切除应答否如卵巢切除 1/3（选 2）。进行手术时时间和年龄至少需要填写一项。

体力活动

请注意：此处填写的为**过去一个月内**平均**每周**的体力活动时间！而**非**平均**每天**的体力活动时间！提问时可以先问受试者有无此项体力活动，然后提问其每天大约花在此活动上的时间，然后计算其一周的总时间。注意：部分活动可能平时和周末会有区别！

为保证体力活动问题回答的准确性，相应问题的定义列在表格下。为便于参考，重、中、轻三级体力活动例子见**表 6.1 不同强度体力活动分级、举例**。

表 **6.1** 不同强度体力活动分级、举例

重度（**6.0-12.0METs**）	● 田间劳动（翻地、犁地、刨地）<br>● 扬场<br>● 打扫牲口圈、养鸡/鸭场<br>● 劈柴	● 修路<br>● 装卸<br>● 搬运重物<br>● 用锹、铲挖掘<br>● 建房<br>● 搬家俱	● 跑步<br>● 打网球<br>● 自由泳<br>● 疾走<br>● 跳绳<br>● 篮球比赛<br>● 举重
中等（**4.0-5.5METs**）	● 做面点（包）<br>● 教体育课<br>● 园林、农田浇水<br>● 驾驶货车、拖拉机、大客车<br>● 饲养家畜、家禽、宠物<br>● 割草	● 骑自行车<br>● 迪斯科舞、民族舞、体操<br>● 打乒乓球、排球、羽毛球<br>● 打太极拳<br>● 健身俱乐部锻炼	● 照看儿童、老人、残疾人<br>● 收拾庭院<br>● 爬楼梯
轻度（**1.0-3.8METs**）	● 讲课<br>● 警察<br>● 实验室工作<br>● 修鞋、修锁、缝纫	● 小型机动车驾驶（轿车、摩托车）<br>● 售货<br>● 演奏乐器	● 一般家务劳动（如搞房间卫生）<br>● 做饭、准备食物<br>● 拖地<br>● 养花种草
步行	● 步行上下班<br>● 购物	● 推婴儿车<br>● 散步	● 遛狗
静坐	● 安静地坐着书写、读书、交谈<br>● 办公室工作	● 安静地坐着看电视、吸烟、听音乐<br>● 编织	● 操作电脑（打字、画图、上网、游戏等）<br>● 宗教活动（如上教堂）<br>● 下棋、打牌、麻将

填表时在最后选择方框内填答案，如 **20** 题 **A** 回答没有跑步、游泳活动时，在方框内选择填写“无”所对应的数字“1”，如进行过此类体力活动，则需要填写相应的时间所对应的数字。老年人

应关注他们每天家务劳动的持续时间。

20 题 L 中步行时间填写应估计平均每天的步行时间，再乘以“7”得到一周全部步行时间，如每天上下班步行时间共为 20 分钟，工作中步行时间共为 40 分钟，采购步行 30 分钟，则一周总时间为 10.5 小时，填写相应的数字“6”(但如果每周只有 5 天工作时间，则选择“5”)。**注意：**L、M、N 中都为步行，但定义不一样。请看清题意，并提醒受试者注意。

照看儿童、老人、残疾人，饲养宠物等要区分其活动时间以及坐立时间。活动时间算作中等强度体力活动中，而坐立时间则算作在家中坐立时间。应仔细分别询问！

以下药物使用登记和膳食信息为单独表格，由专人询问和记录。

<u>药物使用登记</u>

1 此处填写目前正在常规服用的药物名称，包括糖尿病药物及治疗高血脂、高血压等的药物。最好可以让受试者把服用的药物标签或说明书带来，方便填写。

2 此处填写目前服用的保健品。如维生素片、膳食纤维、钙片、各类市场销售的保健品和营养补充剂。

<u>膳食信息</u>

1 询问**过去一年中**以下食物的食用次数和数量填写在下表中，在次数前打√，并填写食用数量(**指生食，净重**)。

<u>询问方式：</u>

豆类及其制品

A, 吃黄豆、大豆或黑豆吗？每月吃几次？每次吃几两？

B, 每周吃几次豆腐（豆制品）？每次吃几两？

C, 喝豆浆、豆腐脑(豆腐花)吗？平均每周（每月）喝几次？每次喝几碗？

D, 吃黄豆粉吗？多久吃一次？每次吃多少？

E, 吃豆芽吗？包括黄豆芽和绿豆芽。每月吃几次？每次吃几两？

F, 喝豆腐花 (豆腐脑)吗？平均每周（每月）喝几次？每次喝几碗？

G, 除去豆腐，豆浆外，其他的豆制品（如豆腐干、百页、素鸡、腐竹、豆腐皮、油豆腐、豆奶粉等）经常吃吗？每周（天）吃几次？每次大概吃几两？

H, 除去黄豆、大豆，其他的豆类（如蚕豆、扁豆、毛豆、豇豆、绿豆、赤豆、芸豆等）经常吃吗？每月（周）吃几次？每次大概吃几两？

I, 经常吃豆豉/黄酱/豆瓣酱/腐乳吗？每月吃几次？每次吃多少？

其他

J, 杂粮(小米、玉米、燕麦、荞麦、高粱等)这些经常吃吗？多久吃一次？一次吃几两？

K, 坚果类的食物（如花生/瓜子/核桃/南瓜子等）多久吃一次？一次吃几两？

L, 芝麻经常吃吗？多久吃一次？一次吃多少？

M, 喝红茶，绿茶吗？平均每月（每年）用几两茶叶？

N, 常吃水果吗？平均多长时间吃一次，每次吃几个？大概有多大多重？

2．通常全家几口人一起用餐？（记录就餐人口数）

食用油

家里面食用油主要是用哪个品种的？

A, 植物油全家每月用几斤？

B, 用猪油、黄油？（回族不问）全家每月用多少？

C, 用沙拉油、沙拉酱吗？全家每月用多少？

D, 除了以上的，家里面还用其他的食用油吗？每个月用多少？

三、三天膳食记录表格

亚麻子健康改善项目

（三天膳食记录）

编号：____________________

姓名：____________________

住址：____________________

电话：____________________

上海市科学技术委员会项目

中国科学院上海生命科学研究院营养科学研究所

上海华东医院

2006 年 1 月

填表说明： 为了了解您的饮食营养是否合理，请在体检前一周内任意选择三天记录每天吃的食物，但**必须包括一个周末**。推荐记录周二、周四、周六三天的饮食摄入。请不要选择外出就餐的一天记录。请按照餐次和种类记录，选择相应吃的食物，在其后填写重量。若没有吃该种食物，请在名称或重量处写“0”作为记号。若表中没有您所吃的食物，可在“**其他**”栏中，填写食物**名称**及**重量**。若空间不够，可在旁边空白处填写。

日期：2006 年______月______日/ 星期______

餐次	食物种类	名称 / 重量	名称 / 重量	名称 / 重量
早餐	乳类	牛奶 ______袋/盒<br>豆浆 ______袋/盒	酸奶 ______袋/盒<br>豆奶 ______袋/盒	豆腐花 ______盒<br>水果汁 ______毫升
	主食	面包 ______片/只<br>菜包 ______个<br>油条 ______根	蛋糕 ______只<br>肉包 ______个<br>8、______ ______个	稀饭 ______小碗<br>豆沙包 ______个<br>9、______ ______只
	蛋类	鸡蛋 ______个	咸蛋 ______个	3、______ ______只
	佐菜	肉松 ______两	酱菜 ______两	3、______ ______两
	其他	1、______ ______	2、______ ______	3、______ ______
中餐	主食	米饭 ______小碗<br>水饺 ______只	面条 ______两<br>5、______ ______个	馄饨 ______只<br>6、______ ______个
	肉类	猪肉 ______两	牛肉 ______两	3、______ ______两
	家禽类	鸡肉 ______两<br>鹌鹑 ______两	鸭肉 ______两<br>5、______ ______两	鸽子 ______两<br>6、______ ______两
	鱼类/海鲜	1、______ ______两	2、______ ______两	3、______ ______两
	豆制品	1、______ ______两	2、______ ______两	3、______ ______两
	蔬菜类/鲜豆类	1、______ ______两<br>4、______ ______两	2、______ ______两<br>5、______ ______两	3、______ ______两<br>6、______ ______两
	饮料类	1、______ ______杯	2、______ ______杯	3、______ ______杯
	其他食物	1、______ ______两	2、______ ______两	3、______ ______两
晚餐	主食	米饭 ______小碗<br>水饺 ______只	面条 ______两<br>5、______ ______个	馄饨 ______只<br>6、______ ______个
	肉类	猪肉 ______两	牛肉 ______两	3、______ ______两
	家禽类	鸡肉 ______两<br>鹌鹑 ______两	鸭肉 ______两<br>5、______ ______两	鸽子 ______两<br>6、______ ______两
	鱼类/海鲜	1、______ ______两	2、______ ______两	3、______ ______两
	豆制品	1、______ ______两	2、______ ______两	3、______ ______两
	蔬菜类/鲜豆类	1、______ ______两<br>4、______ ______两	2、______ ______两<br>5、______ ______两	3、______ ______两<br>6、______ ______两
	饮料类	1、______ ______杯	2、______ ______杯	3、______ ______杯
	其他食物	1、______ ______两	2、______ ______两	3、______ ______两
其它	水果	1、______ ______两	2、______ ______两	3、______ ______两
	点心	1、______ ______两	2、______ ______两	3、______ ______两
	零食	1、______ ______两	2、______ ______两	3、______ ______两
	其他	1、______ ______两<br>4、______ ______两	2、______ ______两<br>5、______ ______两	3、______ ______两<br>6、______ ______两

四、三天膳食记录表格填写说明

该表格是提前发给受试者，并要求受试者在前往华东医院体检（非随访）前一周内任意选择三天记录每天吃的食物，以计算和衡量该受试者的能量和营养素的摄入水平。研究人员应在体检前一周之前电话或面对面通知受试者填写该表格，以及填写的注意事项。记录表格必须包括一个周末，**推荐**（但不强求）记录周二、周四、周六（或星期天）三天的饮食摄入。提醒受试者不要选择外出就餐的一天记录。按照餐次和种类记录，选择相应吃的食物，在其后填写重量。若没有吃该种食物，请在名称或重量处写“0”作为记号。若表中没有您所吃的食物，可在“其他”栏中，填写食物名称及重量。若空间不够，可在旁边空白处填写。受试者若对重量估摸不准，可于体检回收该问卷时询问受试者，并由专门的营养师根据经验判断其重量。

特别提示：特别需要提醒受试者该表格为记录性质，而非回忆性质的。因此，最好于当天饭后记录下所吃食物，以保证准确性。

第七部分、体格检查标准操作规程

注意事项

1. 体检所用设备均由营养科学研究所统一提供；
2. 体检的房间要求安静、明亮，地面平整坚固，室温 20°C 左右；
3. 体重、腰臀围的测量须在清晨空腹状态下进行，应提前通知受试者；
4. 体重、腰臀围的测量要求男女分开；
5. 身高、体重、腰臀围的测量须由 4 名调查员完成；
6. 结果记录要求用正体书写，避免潦草。
7. 各项测量由专人完成。

一、身高

（一）仪器准备

1. 从塑料袋中取出可折叠身高仪；
2. 先拔出滑测板，而后拔出四根身高仪杆，放置在干净的塑料包装袋上面；
3. 将身高仪托板放置于靠墙的平整地面上，将读数最小的杆插入托板的孔中；
4. 根据标示数字从小到大依次插入剩余三根杆；
5. 将滑测板套入杆中；
6. 在身高仪和墙壁的空隙处安放塑料泡沫等硬物，使得身高仪固定。

（二）测量过程

1. 测量前，要求受试者脱去头饰物（帽子、高耸的假发等）、外衣、鞋等。双脚站立在踏板的脚印上，取立正姿势，挺胸收腹，两臂自然下垂，双膝并拢挺直，脚跟、臀部和两肩胛上角间三个点同时接触立柱，两眼平视正前方，眼眶下缘与耳廓上缘保持在同一水平（如图 6.1）；

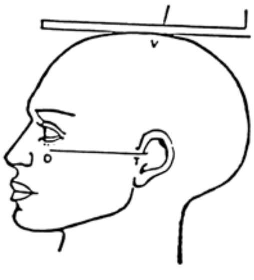

图 6.1 头部位置

2. 开始测量时，一测量员手扶滑测板轻轻向下滑动，直到其底面与颅顶点相接触，然后检查受试者姿势是否正确，确认姿势正确后测量员平视，读取滑测板中间两个黑色箭头所对应的数

字，**以厘米为单位，精确到 0.1 厘米**。测量完毕后结果记录在受试者的《体检表》上；

（三）仪器维护

每处现场测量完成后，将身高仪拆卸装入塑料包装袋并放入塑料盒中。

1. 从身高仪杆中取出滑测板，放于一个安全的地方；
2. 垂直向上将最上面一个杆拔出(切勿左右晃动),并依次拔出剩余三根杆；

   注意：为保持杆与杆之间接头完好无损，请务必从最上面一个杆依次拔出所有杆。
3. 将四根杆倒插入身高仪的托盘中，再将滑测板插入杆和托盘之间；
4. 将身高仪装入塑料袋并放入塑料盒中。

二、体重

（一）仪器（**Seca-882**）准备

1. 电子体重计由体重仪和读数仪两个部件构成，由一根 2 米的连接线连接。两个部件必须同时从纸箱中取出，将体重仪放置在坚固的水平地面上，并将读数仪放在体重仪后面的桌子上；注意连接线的放置，避免绊人。
2. 小心翻转体重计，找到背面“电池”标志，按箭头指示的方向，拨动卡口，电池盖自动打开，小心取出整套电池，将电源导线接头和电池盒接头连接，重新放置好电池盒，合上盖子，翻转体重计，重新放置在坚固的水平地面上；
3. 按读数仪上的绿色“START”（开始）按钮，当读数器显示 0.0kg，表示电源连接正常，体重计可以使用。

   (如果显示为 0.0lb（0.0 磅），轻按“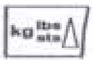”键进行切换至 0.0kg（0.0 公斤），如果显示 0:00.0sts，长按“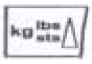”键进行切换至 0.0kg)

   注意：本仪器还有其他功能，但在本次体检过程中，一般情况下只需使用“START”键，勿按其他键；一旦不小心按了其他键，可按“START”键退回到体重测量功能。

（二）测量过程

1. 测量前，要求受试者脱去鞋帽和外衣，仅穿贴身内衣；（如除内衣外还有其他衣服，按照提供的标准衣服重量表格进行扣除）
2. 待体重计归零后，受试者平静站于踏板的中央，身体取立正姿势，两眼平视前方，两手放松，自然下垂，掌心向内置体侧。当读数稳定时读取数值，**精确到 0.1 公斤**。将结果记录在《体检表》上。

（三）仪器维护

1. 每处现场测量完成后将体重仪和读数仪放入运输盒内，并确保读数仪液晶显示屏的安全；
2. 装载运输过程中避免剧烈晃动；
3. 使用过程中避免导线等接触热的物体；
4. 使用干净的毛巾或纱布清洁体重仪，用面巾纸擦拭读数仪液晶显示屏。

（四）常见问题及解答

- 测量时无数据显示？

——按“START”按钮

——检查电池的连接

- 读数仪液晶屏呈黑色？

——检查连接线是否连接良好

- 测量前显示的不是 0.0kg？

——重新按“START”按钮（此时体重仪上应无任何负重）（其他解决方法见仪器准备）

- 测量前显示“----”而非“0.0”？

——重新按“START”按钮（此时体重仪上应无任何负重）

- 液晶显示仪有一或多格持续亮或者持续不亮？

——相应的显示单元格出问题，联系现场负责人

- 读数仪显示 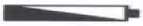 ？

——电源不足，应同时更换 4 节 AA 1.5V 电池

- 读数仪显示 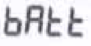

？

——检查电池盒连线；

——未放电池，打开体重仪背面电池盒，放入新电池

- 读数仪显示 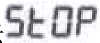

？

——超过了最大负荷

- 读数仪显示屏不断闪烁？

——要求受试者暂时离开体重仪，按“START”按钮两次，直到 0.0kg 显示

- 读数仪显示 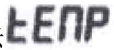

？

——环境温度太高或太低，请在+10°C至+40°C范围内使用。环境温度改变很大时，等待 15 分钟后使用

- 读数仪同时显示 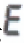

和数字？

－－重新按“START”按钮。如果继续出现同样的情况，通知现场负责人

三、腰围

1. 在只穿内衣情况下进行测量，要求受试者身体直立，腹部放松，两臂自然下垂，双足并拢，两腿均匀负重；
2. 两测量员分别坐或立于受试者前后方，以腋中线肋弓下缘和髂嵴连线中点的水平位置为测量点；
3. 将皮尺轻轻贴住皮肤，经过双侧测量点，保持皮尺与地面平行，勿压入软组织，在受试者平静呼气时读数。**以厘米为单位，精确到 0.1 厘米**，记录在《体检表》上。

    注意：测量完毕后请轻按尺盘中央的按钮，收回卷尺；为保持卷尺的精度，不要随意拉伸卷尺。

四、臀围

1. 在只穿内裤情况下进行测量，受试者身体姿势要求同腰围的测量；
2. 两测量员分别坐或立于受试者的侧方，测量时找出受试者臀部的最大伸展度后，将皮尺环绕臀部一周，保持皮尺与地面平行，贴住皮肤，但避免紧压，然后进行读数；
3. **以厘米为单位，精确到 0.1 厘米**，测量完成后将测量值记录在受试者的《体检表》上。

    注意：测量完毕后请轻按尺盘中央的按钮，收回卷尺；为保持卷尺的精度，不要随意拉伸卷尺。

五、血压、脉搏

（一）测量的要求

1. 环境要求：

    ——室内环境应保持安静和明亮，气温适度（理想的温度应在 20℃上下）

2. 受试者：

    ——避免穿过紧的衣服

    ——测量前 1 小时内应避免进行以下活动：剧烈的运动或锻炼，进食、喝饮料(水除外)，特别是含咖啡因的饮料，长时间暴露于过高或过低的温度下，服用影响血压的药物等

    ——测量前 15 分钟应停止吸烟，精神放松，排空膀胱，安静取坐位休息 5 分钟

3. 测量员：

    ——态度和蔼，避免引起受试者激动

（二）测量过程

测量时应关闭手机、电视机和微波炉等，以免电磁场的干扰，影响测定结果的准确性。

使用前将电子血压计平放在桌面上，取出袖带并将通气导管一端插入血压计底座左侧的接口内，插入后确认接口处密封良好，无漏气。

1. 受试者取坐位，双足平放于地上，感觉舒适即可，脱去外套及右胳膊的内衣衣袖，然后将胳膊放在桌面上，手掌向上，全身放松；
2. 将血压计的袖带缠绕并固定在受试者的右上臂，松紧适度，以可以插入一个食指为宜，袖带中心的高度约和受试者心脏水平位置相齐，袖带的通气管方向朝下，袖带下缘应高于肘关节前肘窝上方约1.3厘米，以使袖带下缘的绿色带段覆盖肱动脉；
3. 正式开始测血压前，嘱受试者在测试过程中保持安静并且避免身体移动；
4. 按下血压计ON/OFF按钮，当符号和“0”同时出现时，表明血压计调零完成；此时按下START按钮，血压计的微压泵开始自动对袖带充气，并同时在屏幕上显示充气值，当袖带内的压力达到最高充气压后（通常较预期的收缩压高30-40 毫米汞柱），袖带开始以每秒2-5 毫米汞柱的速度均匀自动放气，并同时在屏幕上显示放气值。充放气过程中符号闪烁表明正在测定脉搏；
5. 当测量完成时，袖带内的残留空气将被自动排空。显示屏上自动显示收缩压（Systolic/mmHg），舒张压(Diastolic/mmHg)和脉搏(Pulse/min)的测定数值。将测量值记录在被测对象的《体检表》上。

   注意：当由于某些意外情况要中止血压测量时，直接按下ON/OFF按钮，血压计将立即停止充气并开始放气直至关闭。若怀疑受试者的血压高于220mmHg，持续按住START按钮直至显示的充气压高于受试者可能血压的30-40mmHg。

6. 解开袖带，让受试者活动一下胳膊，而后重新缠绕袖带，要求同前。按下Start按钮，进行第二次测量，将结果记录在《体检表》相应栏上；
7. 重复第6条操作，测量第三次血压。注意：每次读数完毕后无需按ON/OFF按钮，即不必关闭血压计。
8. 血压计用后放置5分钟，电源会自动关闭，也可即时按下ON/OFF按钮关掉电源；
9. 当进行重复测量时，因臂部过分充血，会影响测定结果的准确性，如无法保证前后两次测量间隔时间足够长，可嘱受试者上举右臂，缓解肢体的充血程度。

   注意：本血压计有自动存储测量结果的功能，为避免记录混淆，本项目禁止使用存储功能，务必将每次测量的血压结果立即记录在体检表上。

（三）血压计的维护

电子血压计采用精密部件制造，不用时将血压计妥善保存在专门的盒子内。在使用和保管过程中应注意：

- 避免高温、强阳光直射、震荡和灰尘等异常不良环境因素的影响；
- 要避免淋雨、汗水和各种液体污染血压计和袖带；
- 定期用柔软洁净干布（切勿用清洁液、酒精、汽油等液体）对血压计进行清洁；
- 禁止持续按START按钮使充气值高于280mmHg；
- 避免用力折叠袖带和充气管；
- 不必清洁袖带。

（四）常见问题及处理方法

- 开启电源开关后，显示屏上并无显示？

  ——可能电池用尽或放置不正确,请打开血压计后背的电池盒，更换全部电池或调整电池的电极方向。

- 显示屏显示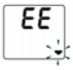?

  ——袖带充气不足，可先关闭血压计，确认导气管是否连接良好，等 2－3 分钟后，重新按下 ON/OFF 按钮进行测量，如果再次出现，可能受试者血压太高，可持续按 START 按钮，直到充气值高于怀疑血压值的 20-30mmHg；如果受试者不可能高于 220mmHg，则可能是袖带包裹太紧，可重新缠绕袖带再测量。

- 显示屏显示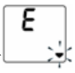 ？

  ——测量过程中受试者手臂移动，要求受试者保持安静不动，重新测量。

- 显示屏显示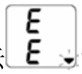?

  ——袖带充气过度（大于 300mmHg），关闭血压计，按标准测量步骤重新测量。

- 显示屏显示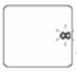?

  ——电池不足，须同时更换为 4 节全新的 AA 碱性电池。

- 显示屏显示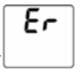?

  ——存储功能失常造成，联系项目办公室（尽管禁止使用存储功能用于本项目，但一旦发现出现此错误信息，应立即联系项目办公室）。

第八部分、血样采集标准操作规程

一、准备工作

（一）知情同意书

受试者需要在知情同意书上签字后方能对其进行抽血。

（二）询问禁食情况

在采血前应询问受试者禁食小时数(需空腹 10 小时左右，如未空腹，不需要采集血样，可改天重新体检)，并填入体检表。另外，为了保证受试者的健康，防止低血糖事件的发生，所有血样的采集应该在清晨 7 到 9 点之间进行。

（三）器材与准备

采血器材：无菌手套、止血带、抽血枕、持针器、酒精棉球、干棉球和废针头处置容器等静脉穿刺所需的材料（与真空采血管配套的一次性蝶形采血针、含 EDTA 抗凝剂的 5ml 紫色头真空采血管以及加入促凝剂的 5ml 红色头真空采血管在受试者采血管袋内，另有多余的备用）。

血液分离、运输及贮藏所需器材：Eppendorf 低温离心机、试管架、1ml 数字移液器、吸头、塑料巴氏吸管、两种贴有编号的受试者采血管袋（分别为分离血浆和分离血清使用，分别含 10 个/人血样冻存管和 6 个/人的血样冻存管）、冰箱。

测量血糖器材：血糖测定仪、测血糖试纸、塑料膜、纸巾、废物桶。

潜在的风险及特殊的防范：

静脉穿刺常见的风险主要有三种：

一是在穿刺部位出现血肿和炎症；

二是出现血管迷走神经反应（包括头晕眼花、出汗、皮肤发冷、手脚麻木、恶心、呕吐、可能的视觉障碍、晕厥及晕厥后跌伤）；

三是出现可能导致血栓性静脉炎的感染。

因此在进行血样采集时应该采取以下防范措施：

- 择通风透气、温度适宜的房间作为血样采集的场所；
- 用于受试者的所有器材均应经过严格的消毒；
- 止血带结扎时间过长可能引起不适并导致血浓缩，因此其使用不得超过 2 分钟，一般应该在血液开始进入真空采血管时便立即松开止血带；
- 用 70%的酒精棉球对采血点进行消毒后应该等皮肤风干以后再进行静脉穿刺，以防酒精在针刺点产生刺痛感或者其它可能会对试验结果产生干扰的应激状态；
- 内科医生应随时待命以防受试者出现不良反应，现场应备有相应的抢救设施及药品。

二、血样的采集及处理

（一）血样的采集

1. 洗手并戴上手套；
2. 准备好试管架、无菌手套、抽血枕、持针器、酒精棉球、干棉球等；
3. 让受试者于采血台前坐下；收取受试者的体检表和血样采集袋（每人两袋，分别装有血样信息表、真空采血管、蝶形针、10 个/小袋和 6 个/小袋的冻存管），认真核对其姓名和编号，询问并登记禁食时间（未经过禁食的受试者不能用于血样采集）。简要解释接下来需要进行的抽血过程，让受试者准备伸出左手臂进行血样的采集（采血尽量从未经测量过血压的手臂上进行）；
5. 打开采血管袋并取出贴有编号的真空采血管和血样信息表、蝶形针，将两只采血管袋递给不同的分血员。分血员将冻存管（10 个和 6 个）取出并依次排列在试管架上；
6. 打开针包但不要去掉针头套，将针头固定在持针器上。将贴有受试者编号的真空采血管（先采集血浆，故先使用紫色头采血管采集血样）插入持针器内，但是不要让针刺破采血管的橡皮管盖，放置旁边备用；
7. 让受试者脱去任何可能压迫手臂的紧身衣物，露出上臂，并将其手臂放置于抽血枕上；
8. 结扎止血带于受试者的上臂，通过检查和触摸选择合适的静脉血管，然后消毒针刺点；

   注意：消毒后不应再触摸针刺点，使针刺点自然风干。
9. 去掉针头套，手持蝶形针的针翼进行静脉穿刺（穿刺时让受试者的手臂向下，以防止血液倒流），发现回血则表明静脉穿刺成功，然后将真空采血管略微用力向前推动，使固定在持针器内的针头刺破采血管橡皮管盖。一旦采血管的管盖被刺破，由于采血管内外的压力差，血液将自动地抽吸到管内。

   注意：穿刺时应该将针的斜面向上沿着静脉血流方向快速而平稳地刺入静脉，避免因为血管损伤所产生的溶血现象。
10. 止血带的结扎不得超过 2 分钟，一旦血液进入采血管就立即松解止血带，以便减少血液浓缩。采血时，采血管的管盖应该高于管底水平，尽量不要让管内的血液和 EDTA 接触到塞子或针尖，防止血液倒流。

    注意：1. 采血过程中如果出现静脉塌陷，应立即停止操作，然后尝试另一手臂。如果仍然失败就应该终止对该受试者的采血，并在《血样信息表》上登记“未采血”。

    2. 如果第一次抽血未达到需要血量，或者采血管出现异常不能自动抽血，则需换上备用的真空采血管，并在采血结束时立即从受试者的采血管袋中取出备用编号贴上。

11. 当血液达到采血管规定的容积后会自动停止流入，这时将采血管从持针器上取下。
12. 立即换上红色头采血管，采集血样至规定的容积后将采血管从持针器上取下。
13. 然后将装有血液的含有 ETDA 抗凝剂的紫色头采血管上下颠倒 8～10 次，使抗凝剂（EDTA）与血液充分混合，但不能剧烈晃动以免出现溶血。将装有血样的采血管暂放置于 4℃冰箱内保存，等待离心分装。
14. 将装有血液的含有促凝剂的红色头采血管立即上下颠倒 5～6 次，使促凝剂与血液充分混合，但不能剧烈晃动以免出现溶血。交给相应的分血员，静置于室温等待其自然凝结（约 30 分钟）。
15. 将针头从静脉拔出，用干消毒棉球压迫针刺点止血，如果出血不止则可用绷带。盖上蝶形针套，将针头递给化验员，用残留于蝶形针硅胶管内的血液进行血糖测定（见后面 **"血糖的测定"**部分）。
16. 再次确认采血管的编号与体检表上的编号一致，在血样信息表上登记采血异常情况并签名。将体检表递给化验员，血样信息表递给分血员。准备采集下一个受试者的血样。

（二）血糖的测定

1. 取出强生 Surestep *Plus*（稳步倍加型）血糖测定仪，安装好电池；
2. 调校血糖仪显示的试纸代码：打开血糖测定仪，显示区会显示上一次的测试结果，稍候出现仪器代码。每次打开新的一瓶试纸条时，须确定血糖测定仪显示的代码是否与试纸瓶上的代码否相符，按 C 按钮调校代码，直至血糖测定仪及试纸的代码相符为止；
3. 取出一条试纸，用试纸的粉红测试区沾取塑料膜上的血液滴，反转试纸，检查"血量指示圆点"是否完全变为蓝色；

   注意：1.务必使血滴接触在试纸条的粉红测试区内，两侧的白色吸收垫可将额外的血液吸收。注意不可直接将血滴靠在白色吸收垫上。血量指示圆点位于试纸背后，用以确定血样是否适量。

   2.若在蓝点上出现白色斑纹表示血量不足，必须用新的试纸再测试，以免出现错误的结果。若滴入过量血液渗满整个白色吸收垫，必须用新的试纸再测试，以免出现错误的结果。
4. 将试纸插入（粉红测试区向上）血糖测定仪，平均 30 秒得出准确结果；

   注意：滴血后 1 分钟内必须将试纸插入血糖测定仪，否则血液会凝固，影响测试结果。
5. 当血糖测定仪出现清洁提醒时，按下列步骤进行清洁：按着试纸支撑区的顶部，将其退出。用沾蒸馏水的棉签或软布清洁试纸支撑区的两面，然后擦干。同法清洁感光区及感应器，并用软布擦干。然后将试纸支撑区放回原位。

（三）血样的离心和分装

装有抗凝剂的血样（紫色管）须在抽血后 15 分钟内在现场进行离心分离，装有促凝剂的血样（红色管）在自然凝结之后在现场进行离心分离，然后将血浆、白细胞、红细胞和血清分别分装入不同的冻存管内，最后放入-20°C的冰箱内暂时保存并于当天转运至-80°C冰箱内保存。

血浆的处理步骤：

1. 将 Eppendorf 5702R 台式低温离心机安放在平稳的桌子上，安装好转子、吊篮和适配器。最好在安装后放置 2 小时再接通电源开机，以保护压缩机；
2. 按 temp▲▼键将离心机的工作温度调整为 4°C，按下 speed 旋钮可以选择 rcf，旋转 speed 调整转速为 2600g，旋转 time 旋钮调整离心时间至 10 分钟；

   注意：在离心机盖打开的状态下如同时按下 open 和 short 键 5 秒以上，离心机的参数就被锁定以防止无意的调整，time 和 speed 旋钮将不能旋转，只能下按。重复上述动作可以解锁。
3. 关好盖子预冷至少 20 分钟。如果来不及充分预冷，可以按下 fast temp 键，转子便能快速到达设定的温度；

   注意：如果尚未达到预设温度，并有大于 3°C的偏差时，离心机在运行将会发出信号。
4. 当离心机的工作温度降到 4°C后，把装有血样的采血管从 4°C冰箱里取出，按 open 键打开离心机盖，将采血管依次对称放入离心机的吊篮内，保持平衡；

   注意：1. 如果一次离心的采血管数为单数，或者有个别采血管不满，需用准备的白盖采血管作为平衡管。平衡管内加入清水的体积应为对称采血管内血液体积的 1.3 倍。

   2. 5702R 离心机有一个自动的不平衡探察器，如果重量差异过大，会自动停止离心，并显示错误信息“inb”
5. 盖好离心机盖，向下按 time（start/stop）键，离心机开始工作。开始运转时会有一个实心方块的标志持续闪烁；
6. 在离心的同时，检查试管架上冻存管的编码是否一致；
7. 到达设定的时间（15 分钟），离心机会自动停下，并发出“嘀嘀”信号指示离心已经完成。打开盖子，将采血管从离心机内取出，并依次摆放在试管架中相应的空位上，按编号对应每组的 10 个冻存管，切忌放错位置；
8. 从试管架的第一个采血管开始分装。将 1ml 的移液器调节到 500µl，然后轻轻打开采血管的盖子，切忌用力过猛，将已经分层的血样搅动。用移液器将上层的血浆轻轻吸出分装到前 6 个冻存管内(管盖颜色分别为：红色、橙色、黄色、绿色、棕色和紫色)。余下的血浆全部装到

第 6 管紫色管内，**一定不要把下层的白细胞层吸出**；

9. 取出血浆后，用一次性塑料吸管将白细胞层 (界于血浆层和红细胞层之间) 轻轻吸入到一个管盖颜色为白色的冻存管内（大约 400~500μl）。为保证白细胞量充足，少量吸入底层的红细胞没有关系；
10. 更换新的吸管将底层的红细胞吸出并分装入剩下的 3 个粉红色管盖的冻存管内（大约 1000μl/管）。将使用过的采血管、管盖、塑料吸管丢入特定的废物桶内；
11. 把所有的冻存管的盖子盖上并拧紧，未装满的冻存管在“血样信息表”的相应位置画“╳”，分血员签名，然后一起装回塑料袋内并放入-20°C以下的冰箱内保存；

    注意：每一人份合格血样必须包含至少四管血浆（每管体积不得少于 400μl），一管白细胞（每管体积大约 400-500μl）和三管红细胞（每管体积大约 800μl）。
12. 进行下一份血样的分装，重复步骤 8-11；
13. 每天使用结束时用柔软的吸水性强的毛巾擦去转子腔体内的凝结水，关闭电源。定期清空和清洁离心机左侧的凝结水收集器；

    注意：如果遇见离心管破裂情况，为彻底清洗，取出离心管适配器和圆形吊篮并分别用清水、酒精等溶剂清洗各部分。不要使用含有次氯酸钠、氯化钠或氧化剂的消毒剂或清洁介质。
14. 离心机运输时需将吊篮、转子和适配器取下单独包装，以免震动损伤配件。

血清的处理步骤：

血清采集管内加入了促凝剂。基本操作步骤和血浆分离相似。主要的区别在于：血样采集后，上下颠倒 5～6 次，使促凝剂与血液充分混合，静置于室温中。待其自然凝固分层后（约 30 分钟），放入 4°C离心机中离心，2600g 离心 10 分钟。离心结束之后，把采血管从离心机中取出。使用移液器将上层的血清轻轻吸出分装到前 6 个冻存管内(管盖颜色分别为：红色、蓝色、黄色、绿色、白色和灰色，500ul/个)。余下的血清全部装到第 6 管灰色管内。下层血细胞无需保存，丢弃至特定的废弃物收集箱。分装后填写分血员姓名，立即放入-20°C以下的冰箱内保存。

（四）血样转运到上海市营养科学研究所中心实验室

转运前，请务必查清血样是否标好编码，每 10 个临近号码的采血管袋装入一个大袋，并随包装箱附上货运清单，以便及时清查可能遗失的血样。血浆和血清分开装袋，并在袋子外面注明。

运输方法：所有的样品（包括血清、血浆，白细胞，红细胞）必须使用冰块运输，确保血样处于冻存状态。运输过程中要注意防止血样的融化。

血样转运箱内须附带的文件：货运清单及若干完整的采血管袋，内含十（或六）个冻存管、血样信

息单【样品的编号、血量（ml）以及血清、血浆、白细胞和红细胞各分装的管数以及采血员、分血员的姓名】。

注意：运输时箱子内应留有适当多余的空间，以防热胀冷缩效应导致箱子破裂。另外，要尽量缩短血样的运输时间（最好在 12 小时之内），确保血样一直处于低温保存状态。

血样的复核验收和储存

在收到血样后，应该立即打开血样包裹箱，根据附带的货运清单核对样品和相关的资料。然后将血样冻存于-80°C 冰箱中长期保存。

三、血样采集过程的安全事项

（一）实验室操作安全

不允许在实验室吃东西，实验室台面应保持清洁，每天用消毒液擦拭。一旦有血液溅落，即用 75%酒精或消毒液擦拭。采血及处理血样的过程均要求戴无菌手套。

（二）针头等材料的处理

所有的采血员都要配备特定的废弃物收集箱。每次采血结束后应该将用过的针头、酒精棉球、生化分析废弃物、分离血样用的吸嘴吸管、采血管等物品丢入废弃物收集箱，严禁回收或者重复利用。此外，废弃物收集箱不能装的太满，以免引起危险。每天操作结束后，统一集中处理生化废弃物。

（三）针头刺伤

在进行静脉穿刺时要防止被针头刺伤。如果不幸被针头刺伤，应该按照以下急救方案进行处理：

- 保持镇定，避免恐慌，防止再一次被刺伤；
- 用大量清水冲洗被刺伤处，切勿挤压；
- 将含 70％酒精的棉球置于伤口上放置两分钟，以达到消毒的目的；
- 与当地负责处理疾病传染的专业人员联系，并听从其指导。

表 3 血样转运清单

血样数量（人份）		血样信息表（人份）		
是否冰块运输	是		否	
发货时间	2006 年 月 日 时 分	收货时间	2006 年 月 日 时 分	
货运负责人		收件人		
其它				

表 4 血 浆 血 样 信 息 表

编 号			说 明*	
血 浆	管盖颜色	体积（μl）	备 注*	存 放 位 置
	红色	500		
	橙色	500		
	黄色	500		
	绿色	500		
	棕色	500		
	紫色	500		
白细胞	白色	600		
红细胞	粉红色	1000		
	粉红色	1000		
	粉红色	1000		
采血员			分血员	

*说明：如有必要，填写“溶血”、“乳糜”及“不足”等总的血样信息。

*备注栏：血样没有时，在相应栏打“X”；不足时，在相应栏估计体积写上。

表 5 血 清 血 样 信 息 表

编 号			说 明*	
血 清	管盖颜色	体积（μl）	备注*	存 放 位 置
	红色	500		
	黄色	500		
	蓝色	500		
	白色	500		
	绿色	500		
	灰色	500		
采血员			分血员	

*说明：如有必要，填写“溶血”、“乳糜”及“不足”等总的血样信息。

*备注栏：血样没有时，在相应栏打“X”；不足时，在相应栏估计体积写上。

第九部分、尿样收集标准操作流程

一、准备工作

（一）发放储尿瓶及说明

提前发放给每位受试者储尿瓶以及如何接尿的说明。储尿瓶外提前贴好受试者编码。

（二）电话通知或提醒受试者收集尿样及注意事项

体检前 3 天内电话提醒受试者体检时间、地点和注意事项。特别需要提醒受试者如何留尿以及将储尿瓶带至体检现场。留尿样前应空腹。最好为清晨起床后第一次排尿的中段尿。尿液装满储尿瓶的大部分即可，不要全满。

（三）器材与准备

无菌手套、试管架、贴有编号的 6 个/人的 2ml 尿样保存管、-20°C 冰箱。

二、尿样的采集和处理

（一）接收尿瓶

接收受试者带来的储尿瓶，检查编号，并在体检表和名单相应地方划“√”标注。将储尿瓶交至分尿员进行分装。

（二）尿样分装

分尿员接到受试者的储尿瓶后，应加入约 50mg 的抗坏血酸，充分摇匀。找到对应编号的尿样保存管，排列在试管架上。充分混匀储尿瓶中尿样后，取 1ml 尿样至每个相同编号的尿样保存管中。将分装之后的 6 支/人小管放入有相应编号的小袋中，然后将储尿瓶和小袋都放入-20℃保存。

（三）受试者未带尿瓶的解决办法

若受试者忘记带储尿瓶，或其尿样不符合条件，可让受试者在现场留尿。发给受试者一次性接尿杯，告知受试者接尿方法，安排起到指定地点接尿。收集尿样后，倒入 45-50ml 尿样至储尿瓶中，加入约 50mg 的抗坏血酸，充分混匀。再交给分尿员进行分装。（现场准备有未贴标签的储尿瓶以及编号）

（四）尿样转运到上海市营养科学研究所中心实验室

转运前，请务必查清尿样是否标好编码，并随包装箱附上货运清单，以便及时清查可能遗失的尿样。

运输方法：所有的样品必须使用冰块运输，确保尿样处于冻存状态。运输过程中要注意防止尿样的融化。

尿样转运箱内须附带的文件：货运清单及完整的尿样保存管，尿样信息单【样品的编号、尿量（ml）以及分尿员的姓名】。

注意：运输时箱子内应留有适当多余的空间，以防热胀冷缩效应导致箱子破裂。另外，要尽量缩短尿样的运输时间（最好在 12 小时之内），确保尿样一直处于低温保存状态。

尿样的复核验收和储存

在收到尿样后，应该立即打开尿样包裹箱，根据附带的货运清单核对样品和相关的资料。然后将尿样冻存于-20°C 冰箱中长期保存。

三、尿样采集过程的安全事项

（一）实验室操作安全

不允许在实验室吃东西，实验室台面应保持清洁，每天用消毒液擦拭。一旦有尿液溅落，即用干净抹布或纸巾擦拭。采集尿样及处理尿样的过程均要求戴无菌手套。

（二）吸管等材料的处理

所有的分尿员都要配备特定的废弃物收集箱。每次分装尿液结束后应该将用过的枪头、酒精棉球、纸巾等物品丢入废弃物收集箱，严禁回收或者重复利用。此外，废弃物收集箱不能装的太满，以免引起破裂。每天操作结束后，统一集中处理生化废弃物。

表 5 尿样转运清单

尿样数量（人份）		尿样信息表（人份）	
是否冰块运输	是	否	
发货时间	2006 年　月　日　时　分	收货时间	2006 年　月　日　时　分
货运负责人		收件人	
其它			

表 6 尿样信息表

**编号**			**总尿量（ml）**	
**储尿瓶**	**管盖颜色**	**体积（ml）**	**备注***	**存放位置**
	棕色	40-50		
冻存小管	红色	1.0		
	黄色	1.0		
	蓝色	1.0		
	白色	1.0		
	绿色	1.0		
	灰色	1.0		
**分尿员**				

*备注栏只需要在体积达不到要求的相应栏打“X”即可。

第十部分、质量控制

本研究过程中，应由项目负责人指派的监查员定期进行现场监察询问，以保证临床试验中受试者的权益得到保障，试验记录和报告的数据准确完整无误，保证试验遵循已批准的研究方案和有关法规。参加研究人员必须经过统一培训，使用统一记录方式与判断标准。各实验室检查方法与判断标准必须统一。针对可能发生的受试者的脱落，应积极采取措施，控制脱落率在 20%以内。数据管理员采用 EPIDATA 软件编制数据录入程序，进行数据录入与管理。为保证数据的准确性，采用双份录入并校对。所有实验室生化指标检测每份样本重复测定一次。

质控原则：上海营养所负责项目质量控制方法确定，统一调查方法和调查表；负责整个试验参与人员培训、考核，对体检现场进行技术指导和质控。华东医院负责人负责临床干预的具体实施、负责膳食问卷的质控、与营养所共同完成其他各项质控。项目质控组由双方共同组成。

一、问卷质量控制

1. 上海营养所质量控制组负责一般问卷完成后的质量控制，每次问卷后负责检查是否有漏项、错项，发现有漏项、错项要及时反馈给问卷的调查人员，由调查员向受试者核实，并填写核实后的正确内容，并最后签字。
2. 华东医院质量控制组负责 3 天膳食记录完成后的质量控制，及时向受试者解释如何记录，并核对记录情况。
3. 调查员要认真阅读调查问卷，掌握领会调查内容。在完成调查后必须对自己填写的问卷表作一次全面检查，查看有无漏项、书写和逻辑错误，发现问题及时纠正。在结束该受试者调查前主要检查如下内容：

- 封面是否填写完整，是否有遗漏，尤其是编号、姓名、住址和电话；
- 检查一般问卷、膳食问卷、知情同意书、体检表和体检通知单的编号是否一致；
- 是否存在前后内容不一致的答案，是否有违反逻辑的错误存在；
- 调查员在发现明显是因为自己的疏忽而出现错误时，须重新询问被调查人。

4. 项目质控组在每次问卷收集并初次质控完成后仍需对 10％的问卷进行抽查，检查问卷的填写情况，发现问题应及时与相关调查员交换意见并确定解决方法，同时将结果记录在《**问卷表质量控制检查结果记录表**》上。

二、体检质量控制

(一) 身高、体重、腰围、臀围测量

1．所有体检仪器均由上海营养科学研究所统一提供。

2．所有项目的测量按照统一标准进行，所有研究人员接受统一培训及考核。

3．所有项目的测量由 2 人共同完成，最大限度减少测量过程中操作误差。

4．质量控制组任务：

■体检开始和体检中校正体检仪器和设备；

■在每次体检时检查体检人员工作过程；

■在体检当天审核体检表，对于填写不合理的数据要及时找出原因以纠正；

■如有必要，体检当天对体检人员测定的身高、体重、腰围、臀围结果进行复核；

（二）血压测量

质控员在体检现场要监督血压测量员的工作过程，确保血压测量操作按要求进行。

三、血样采集的质量控制

现场采血人员必须是具有临床医学或流行病学背景的护士或医师，需要接受标准的采血培训，以达到统一和规范操作并经过考试合格后方能参与现场操作。

在准备现场工作之前，采血员须熟知本次研究的目的，采血相关的操作流程和注意事项。此外，负责质量控制的人员应该不定期地访问采血点，以监督采血员，保证其操作与项目设计的要求一致。在采血现场需要特别注意以下几方面：

● 清楚了解受试者的空腹时间并登记于体检表上；

● 每次操作止血带的使用不得超过 2 分钟；

● 采完血以后应该立即将含有 EDTA 抗凝剂的真空采血管（紫色管）轻轻地上下颠倒 8～10 次，使抗凝剂（EDTA）与血液充分混匀；添加促凝剂的真空采血管（红色管）上下颠倒 5～6 次；

● 所有含有 EDTA 的血样（紫色管）必须在 15 分钟以内进行离心分离，然后放入-20℃以下保存；含促凝剂的真空采血管（红色管）颠倒后室温静置 30 分钟后离心分离；

● 进行血样转运时必须放入足够的冰块，以防止血样在中途解冻。

负责血液离心分离的实验室工作人员应该是专业技术人员，熟悉相关仪器设备的操作，熟悉血液的离心分离和血液常规生化指标的分析及其测定流程。所有参加人员须经统一培训。此外，质控人员应该不定期地访问实验室，以便对实验室的工作实施质控。

四、尿样采集的质量控制

在准备现场工作之前，尿样收集员须熟知本次研究的目的，尿样收集处理相关的操作流程和注意事项。此外，负责质量控制的人员应该不定期地访问尿样采集点，以监督尿样收集员，保证其操

作与项目设计的要求一致。

在尿样收集现场需要特别注意以下几方面：

- 清楚了解受试者的空腹时间并登记于体检表上；（采血和留尿两者只需问一次空腹时间即可）
- 尿样收集后应立即加入 50mg 抗坏血酸防腐剂；
- 所有尿样应尽快进行分装，未及时分装的尿样应置于 4℃或冰上，防止其腐坏变质；
- 尿样分装之后应立即放入-20℃冰箱冻存；

进行尿样转运时必须放入足够的冰块，以防止尿样在中途解冻。

第十一部分、伦理学问题

根据 ICH GCP 的原则，在本次临床试验中应充分考虑伦理学问题：

（1）临床试验的实施应依据源于《赫尔辛基宣言》中的伦理原则，同时符合临床试验管理规范（GCP）及现行的管理法规，我们的目的是最大限度的保护受试者的权益，尽量缩小和避免对受试者的伤害。

（2）试验开始前，权衡可预见的风险和不便，并比较每位受试者的风险和社会预计获得的受益。临床试验只有在预期的益处大于风险时才能予以启动和继续。

试验前，我们对这方面的临床研究文章进行了全面的搜索和综述，认为木酚素和异黄酮等植物多酚类物质对于血脂代谢有很明显的改善作用，对于血糖也有一定的改善作用，但研究不多。同时，由于是天然作物，亚麻子的副作用很小。我国西北部地区居民有长期食用亚麻子的历史。国外给予健康成人和癌症患者 50 克亚麻子未发现有明显的副作用；给予健康成人、绝经后妇女、前列腺癌患者每天 16 毫克/千克体重剂量的异黄酮，一个月的干预时间也未发现有明显的临床毒性；加拿大目前正在进行的一项 NIH 资助的临床试验也表明，每天服用 500 毫克木酚素的胶囊没有明显的副作用。根据以上的一些信息，我们制定了每天 360 毫克木酚素的剂量，并且认为这个剂量是安全的，是可以达到实验设计的。试验的最终目的是为了研究广泛存在于谷物蔬菜和水果中的植物木酚素是否具有改善 2 型糖尿病人中糖脂代谢紊乱等作用，从而为今后通过营养干预和膳食结构调整来预防和控制目前我国迅速增长的糖尿病、高血脂等慢性疾病提供科学依据。

（3）受试者的权益、安全和健康应是首要的考虑，并应胜过科学和社会的利益。

首先，我们制定了严格的入选和排除标准，排除可能会发生不良反应或者严重不良事件的患者（如严重肝肾功能不全者、严重心脑血管疾病者等）参加本试验。其次，我们要求受试者在出现不良事件时，立即停止试验用胶囊的服用，并和研究人员联系，由研究人员来评估不良事件是否和试验用胶囊有关，研究人员将根据不良事件的严重程度采取相应的措施。如果由于服用试验用胶囊造成的任何损害，我们将负责检查并赔偿由此造成的治疗费用。在发生严重不良事件时，

研究人员会立即通知所有参与试验的受试者停止服用胶囊，立即通报伦理委员会，并组织医学专家委员会，来判断该事件是否与试验用胶囊有关。如有关，则会立即中止临床试验，并制定相应的补偿和处理措施。如无关，则可以继续进行临床试验，但会对每个受试者进行密切的观察。

（4）伦理委员会

临床试验开始前，试验方案需经过伦理委员会审议同意并签署批准意见后才开始实施。接受伦理委员会提出的修改意见，并尽快做出回应。在试验进行期间，试验方案的任何修改均应通过伦理委员会批准后方能执行；试验中发生严重不良事件时，会立即向伦理委员会报告。

（5）知情同意书

本试验中，研究者和相关研究人员将向受试者说明有关临床试验的详细情况，经过充分和详细的解释试验的情况后获得知情同意书。知情同意书包括了试验目的、试验背景、预期受试者受益、风险和不便，费用和补偿，信息的保密等各个方面。受试者可以在签署前向研究人员咨询任何关于试验的问题。试验完全是自愿的，受试者可以不参加本试验，也可以随时提出退出试验，这些都不会影响其医疗方案以及和医生的关系。受试者的所有信息都将被严格保密。

（6）随机化、双盲和安慰剂对照

本试验为随机化双盲的临床对照干预试验，这是目前国际上临床试验的“金标准”，具有很强的可操作性、客观性和科学性。但是在实际试验中也会遇到一些道德问题。

由于采用的是安慰剂作为对照组，以及受试者无法知道自己接受的是何种干预，所以可能在实际中会有很多的问题出现。研究人员将会向受试者尽量解释双盲和对照试验的科学性，并采取相应的补偿措施（如对受试者进行糖尿病教育和膳食、生活方式等的指导），并密切关注每次体检的结果，避免异常值的出现影响受试者的身体健康。同时，如果受试者由于治疗自身疾病的需要，可以按照一定的程序揭盲，以受试者的安全和健康为第一目标。

第十二部分、试验进度表

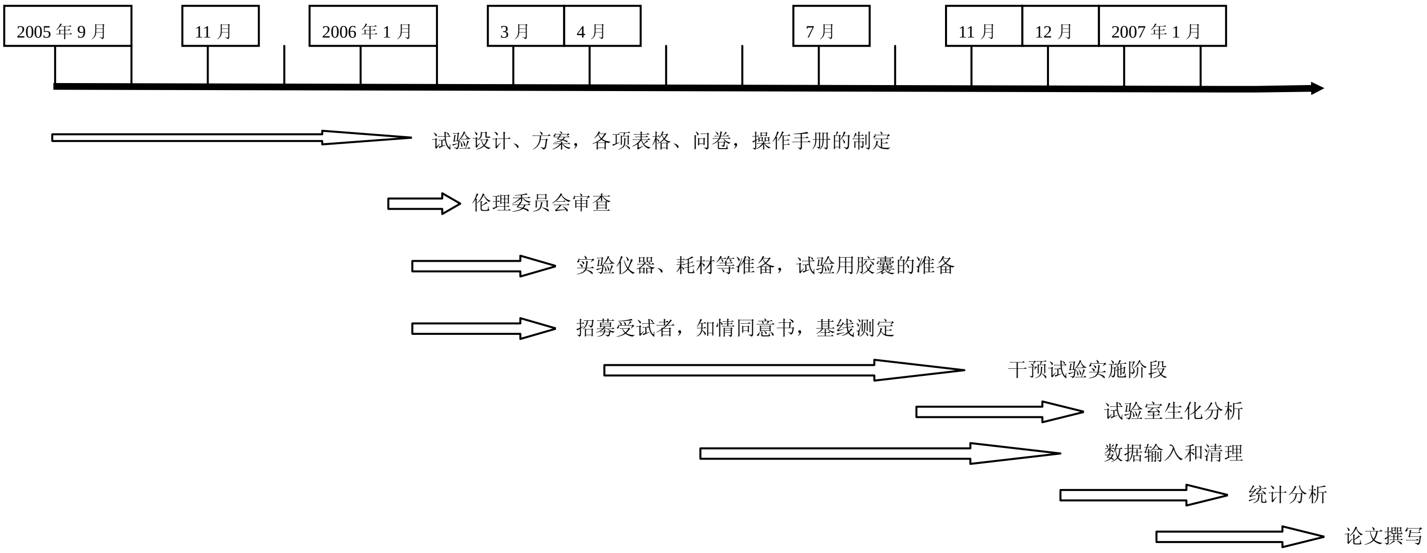

2005.09-2006.01 完成项目标书、调查问卷和现场工作手册的初稿和修改；

2006.01-2006.02 接受伦理委员会的审查；

2006.02-2006.03 招募受试者；

2006.02-2006.04 完成实验室器材、试剂及其他物资订购，试验用胶囊的制备和分装；

2006.02-2006.04 准备培训教材，培训试验研究人员，进行临床试验的前期准备；

2006.03-2006.04 受试者的基线测定，随机化；

2006.04-2006.11 临床试验的干预阶段；

2006.12-2007.01 开展实验室分析工作；

2006.05-2007.03 输入数据和进行统计分析；

2007.03-. 撰写临床试验总结和论文及发表文章。

第十三部分、各方承担的职责及其它规定

营养所主要负责：

（1）试验设计和随机化；

（2）提供招募受试者，试验过程所需的经费（体检，随访，实验室分析，数据处理和受试者补偿）；

（3）提供和发放及回收试验用胶囊；

（4）所有表格（除三天膳食记录问卷）的设计和所有表格（包括膳食问卷部分）的印刷；

（5）一般问卷的调查；

（6）所有数据（除三天膳食记录问卷）的电脑录入（包括体检结果、一般问卷、药物登记、简单

膳食频率问卷和病例报告表等);

(7)提供招募病人和以后现场体检的仪器和设备(离心机,采血设备,尿样收集设备,体重计,身高尺,软尺,电子血压计等等);

(8)共同参与体检。包括:血样分装和尿样分装,部分体格检查,具体的要求见本《操作手册》69-74 页;

(9)参与每三周的面对面随访以及一半的受试者电话随访工作;

(10)所有实验室分析(除筛选病人)。

华东医院主要负责:

(1)筛选受试者。首先进行问卷粗筛,符合条件者再进行抽血,主要分析指标为:血糖、血脂(总胆固醇,甘油三脂,低密度和高密度脂蛋白),胰岛素,肌酐,谷丙转氨酶(GPT);

(2)确定受试者名单,反馈给营养所以进行随机化分组;

(3)为体检和随访提供场地,以及准备体检和随访时受试者的早餐、礼品和现金的补偿;

(4)负责体格检查、血样采集(此处包括了现场血糖测定),以及基线时简单膳食频率问卷、药物登记表格的调查;

(5)在第一和第二阶段干预前后的体检同时负责完成四次 3 天膳食记录的核对检查、录入和分析。负责静脉取血和血糖测试。

(6)每周至少对负责随访的每名受试者进行电话随访一次。每三周对所有受试者进行面对面随访一次,随访内容包括:血压测定、血糖测定,询问其身体状况,回收未服用完的胶囊,发放新的胶囊(后两项由营养所专人负责);

(7)日常管理。

第十四部分、参考文献

Reference List

1. Duncan AM, Phipps WR, Kurzer MS: Phyto-oestrogens. *Best Pract Res Clin Endocrinol Metab* 17:253-271, 2003

2. Kurzer MS, Xu X: Dietary phytoestrogens. *Annu Rev Nutr* 17:353-381, 1997

3. Vanharanta M, Voutilainen S, Rissanen TH, Adlercreutz H, Salonen JT: Risk of cardiovascular disease-related and all-cause death according to serum concentrations of enterolactone: Kuopio Ischaemic Heart Disease Risk Factor Study. *Arch Intern Med* 163:1099-1104, 2003

4. Vanharanta M, Voutilainen S, Lakka TA, van der LM, Adlercreutz H, Salonen JT: Risk of acute coronary events according to serum concentrations of enterolactone: a prospective population-based case-control study. *Lancet* 354:2112-2115, 1999

5. Nagata C, Takatsuka N, Kurisu Y, Shimizu H: Decreased serum total cholesterol concentration is associated with high intake of soy products in Japanese men and women. *J Nutr* 128:209-213, 1998

6. Zhang X, Shu XO, Gao YT, Yang G, Li Q, Li H, Jin F, Zheng W: Soy food consumption is associated with lower risk of coronary heart disease in Chinese women. *J Nutr* 133:2874-2878, 2003

7. Adlercreutz H: Phyto-oestrogens and cancer. *Lancet Oncol* 3:364-373, 2002

8. Adlercreutz H: Epidemiology of phytoestrogens. *Baillieres Clin Endocrinol Metab* 12:605-623, 1998

9. Bierenbaum ML, Reichstein R, Watkins TR: Reducing atherogenic risk in hyperlipemic humans with flax seed supplementation: a preliminary report. *J Am Coll Nutr* 12:501-504, 1993

10. Clark WF, Parbtani A, Huff MW, Spanner E, de SH, Chin-Yee I, Philbrick DJ, Holub BJ: Flaxseed: a potential treatment for lupus nephritis. *Kidney Int* 48:475-480, 1995

11. Jenkins DJ, Kendall CW, Vidgen E, Agarwal S, Rao AV, Rosenberg RS, Diamandis EP, Novokmet R, Mehling CC, Perera T, Griffin LC, Cunnane SC: Health aspects of partially defatted flaxseed, including effects on serum lipids, oxidative measures, and ex vivo androgen and progestin activity: a controlled crossover trial. *Am J Clin Nutr* 69:395-402, 1999

12. Arjmandi BH, Khan DA, Juma S, Drum ML, Venkatesh S, Sohn E, Wei L, Derman R: Whole flaxseed consumption lowers serum LDL-cholesterol and lipoprotein(a) concentrations in postmenopausal women. *Nutrition Research* 18:1203-1214, 1998

13. Lucas EA, Wild RD, Hammond LJ, Khalil DA, Juma S, Daggy BP, Stoecker BJ, Arjmandi BH: Flaxseed improves lipid profile without altering biomarkers of bone metabolism in postmenopausal women. *J Clin Endocrinol Metab* 87:1527-1532, 2002

14. Cunnane SC, Ganguli S, Menard C, Liede AC, Hamadeh MJ, Chen ZY, Wolever TM, Jenkins DJ: High alpha-linolenic acid flaxseed (Linum usitatissimum): some nutritional properties in humans. *Br J Nutr* 69:443-453, 1993

15. Cunnane SC, Hamadeh MJ, Liede AC, Thompson LU, Wolever TM, Jenkins DJ: Nutritional attributes of traditional flaxseed in healthy young adults. *Am J Clin Nutr* 61:62-68, 1995

16. Lemay A, Dodin S, Kadri N, Jacques H, Forest JC: Flaxseed dietary supplement versus hormone replacement therapy in hypercholesterolemic menopausal women. *Obstet Gynecol* 100:495-504, 2002

17. Anderson JW, Johnstone BM, Cook-Newell ME: Meta-analysis of the effects of soy protein intake on serum lipids. *N Engl J Med* 333:276-282, 1995

18. Zhuo XG, Melby MK, Watanabe S: Soy isoflavone intake lowers serum LDL cholesterol: a meta-analysis of 8 randomized controlled trials in humans. *J Nutr* 134:2395-2400, 2004

19. Zhan S, Ho SC: Meta-analysis of the effects of soy protein containing isoflavones on the lipid profile. *Am*

J Clin Nutr 81:397-408, 2005

20. Jayagopal V, Albertazzi P, Kilpatrick ES, Howarth EM, Jennings PE, Hepburn DA, Atkin SL: Beneficial effects of soy phytoestrogen intake in postmenopausal women with type 2 diabetes. *Diabetes Care* 25:1709-1714, 2002

21. Vedavanam K, Srijayanta S, O'Reilly J, Raman A, Wiseman H: Antioxidant action and potential antidiabetic properties of an isoflavonoid-containing soyabean phytochemical extract (SPE). *Phytother Res* 13:601-608, 1999

22. Prasad K: Oxidative stress as a mechanism of diabetes in diabetic BB prone rats: effect of secoisolariciresinol diglucoside (SDG). *Mol Cell Biochem* 209:89-96, 2000

23. Prasad K: Secoisolariciresinol diglucoside from flaxseed delays the development of type 2 diabetes in Zucker rat. *J Lab Clin Med* 138:32-39, 2001

24. Prasad K, Mantha SV, Muir AD, Westcott ND: Protective effect of secoisolariciresinol diglucoside against streptozotocin-induced diabetes and its mechanism. *Mol Cell Biochem* 206:141-149, 2000

25. Cunnane SC, Ganguli S, Menard C, Liede AC, Hamadeh MJ, Chen ZY, Wolever TM, Jenkins DJ: High alpha-linolenic acid flaxseed (Linum usitatissimum): some nutritional properties in humans. *Br J Nutr* 69:443-453, 1993

26. Lemay A, Dodin S, Kadri N, Jacques H, Forest JC: Flaxseed dietary supplement versus hormone replacement therapy in hypercholesterolemic menopausal women. *Obstet Gynecol* 100:495-504, 2002

附录 1 《亚麻子健康改善项目》项目承诺书

1. 研究准则

本项目是由上海市科学技术委员会基金资助的，由中国科学院上海生命科学研究院营养科学研究所（简称**上海营养所**）主持的有关亚麻子中的植物木酚素对 2 型糖尿病人血脂和血糖等体内代谢的改善作用和影响机制的临床研究。本项目作为一项纯学术研究，不带有任何商业性质，不为任何利益集团服务，我们的研究目的是为了探讨广泛存在于植物性食物中的木酚素能否有效地改善 2 型糖尿病患者血脂和血糖紊乱，其研究结果将为今后合理组织糖尿病人的膳食提供科学依据。

研究人员承诺：我们承担的是科学研究项目，为获得预期研究结果所采用的各种研究方法和开展的调查工作均属于科研行为，杜绝一切造假行为和严禁虚假信息。严格保护受试对象的利益，对所搜集的各种信息实行严格保密和控制。

研究人员声明：为研究工作需要采集的全部血液和尿液样本，将全部在国内处理和检测。

2. 临床试验研究准则

在临床试验开始前，必须经过伦理委员会的审查，经过批准后才能进行试验。招募受试者时，要耐心的向潜在受试者解释试验的各个方面，告知受试者可能的受益和风险等，在获得受试者签署的《知情同意书》后，方能开展筛选问卷调查和血液样本采集，符合试验入选标准的受试者才可以进行随机化分组进入正式试验。

研究人员对待受试者要热情，有责任心；耐心解释受试者提出的任何疑问。在问卷调查过程中，应采用尊重受试者的语言和语气，尤其要注意尊重少数民族的风俗和生活习惯。

3. 血样采集准则

（1） 向受试者解释进行静脉采集的潜在风险以及所采集血样的用途，并在获得受试者签署的《知情同意书》后才能对其进行血样的采集。
（2） 在采血点配有内科医生，以防调查对象出现任何不良反应。
（3） 现场准备必要急救设备和药品在需要时使用。
（4） 用于受试者的所有器材均要经过严格的消毒，做好防范措施，尽量避免静脉穿刺中可能出现的风险。
（5） 严格按规定操作，防止血液倒流对调查对象造成的不良反应。
（6） 严格按照国家的相关规定进行针头和被血液污染的物品等生物垃圾的处理。
（7） 实验室作业将严格遵守我国生物实验室操作规章制度。

本研究的临床试验部分将由上海营养所与上海华东医院共同完成。与项目有关的各项活动都将严格遵守我国现行的相关法律、法规和当地政府的各项有关规定。体检结果都将及时反馈给受试者，并给出科学健康指导建议。对于出现的不良事件会立即给予处理，防止严重不良事件的出现。

为保护调查对象个人利益，特设立项目专线电话如下：

中科院营养所：021-54920712 或 54920910　上海华东医院：021-62483180 转 60406 或 60407

中国科学院上海生命科学研究院营养科学研究所

2006 年 1 月

附录 2 知情同意书·知情告知页

亲爱的居民：您好！

中国科学院上海生命科学研究院营养科学研究所和上海华东医院将在上海地区进行一项临床研究项目，其主要目的是研究功能性食物—亚麻子的主要成分植物木酚素对 2 型糖尿病患者血脂、血糖紊乱的改善作用和影响机制。

亚麻子又称胡麻子，在我国主要产自于内蒙古、陕西等西北部地区，当地居民有长期的食用史。亚麻子在西方自古以来用于面包等食品的制作或添加在不同的食品中。近年来，由于对心血管疾病方面的保健作用，亚麻子及其制品在国外重新受到广泛的研究和应用。亚麻子富含ω-3 脂肪酸、膳食纤维、植物木酚素等多种营养成分。国外的很多临床和实验室研究表明，亚麻子及植物木酚素对许多慢性疾病如高血脂、心血管疾病、前列腺疾病、乳腺癌等有改善作用。植物木酚素广泛存在于各种谷物、蔬菜、水果等植物中，但以亚麻子中含量最高。因此，本研究将着重了解是否亚麻子的主要成分植物木酚素能够有效地改善 2 型糖尿病人的血脂和血糖水平，为今后预防和控制糖尿病及高血脂提供科学依据。

您的参与将对本研究有很大的帮助。本研究为双盲交叉试验设计，每个参与者都将服用 12 个星期的植物木酚素胶囊和 12 个星期的安慰剂胶囊（淀粉），以便对照服用前后的疗效。为了防止心理因素的干扰，服用胶囊的顺序对您和研究者双方都是保密的。本试验周期总共为 24 个星期，分为两个阶段。第一阶段为期 12 个星期（4 月初至 6 月底），您需要每天服用 3 颗胶囊。第二阶段同样为期 12 个星期（9 月初至 11 月底），您需要每天服用 3 颗另一种胶囊。中间有两个月（7 月初至 8 月底）的休息时间，这两个月您不需要服用任何研究胶囊，只需要按照原有生活方式生活即可。第一和第二阶段开始和结束时都将有一次静脉采血（采血量为 10 毫升，用于血脂、血糖、糖化血红蛋白和胰岛素等生化指标的分析）和尿样收集（用于亚麻子等代谢产物分析）以及体格检查（身高、体重、血压、腰臀围等），每次体检前需要记录一次 3 天膳食。在每次体检时还将有一份关于个人一般信息、体力活动、食物频率等的问卷调查，以了解您的生活方式和习惯的变化。本着自愿的原则，您可以拒绝回答您不想回答的问题。

为了保证您的身体健康和了解可能出现的变化，您需要在第一和第二阶段的时候每三周和研究人员面对面随访并体检一次，随访时我们将为您检测血糖、血压、体重等指标，并咨询您的身体状况和服用胶囊情况。

本研究全程都有专业医师和营养师参与，并将给您作相关的糖尿病知识教育以及膳食和生活方式的指导。所有的体检结果我们都将及时反馈给您。实验室生化指标如

血糖、血脂、胰岛素、糖化血红蛋白以及整个研究结果将在整个研究工作结束之后尽快反馈给您。在研究过程中，每次抽血参加者将获得 50 元营养费，整个研究中将会有价值 800 元的礼品和现金补偿（分阶段发放）。

亚麻子为天然食物，口感和安全性都很好，其提取物植物木酚素胶囊为纯天然制剂，不含国家禁止的任何添加物，未发现明显的副作用。但在极少数人中可能会出现食物过敏，如果您在服用研究胶囊过程中出现任何以下的症状：皮疹、皮肤瘙痒、面颈部潮红、胃肠胀气、腹泻、恶心、呕吐，请停止服用，并立即和研究人员联系。在研究过程中，如因正确按照说明服用研究胶囊而引起的不良反应，经专家确诊后，我们将对此不良反应的治疗进行赔偿。

您的所有个人信息都会被严格保密。您的姓名不会出现在任何与本项研究有关的公开发表物中如报告、讲座和论文等。本着自愿参与的原则，您可以随时向研究人员提出退出本项目。

本项目纯属科学研究，无任何商业利益和目的。研究经费来自于上海市科学技术委员会和中国科学院。如果您对本项目有任何疑问，可以随时咨询我们的研究人员，或者拨打项目专线（**上海华东医院：021-62483180 转 60406 或 62103，李惠芬；中科院营养所：021－54920712，潘安**），我们将很乐意给您答复。

您可以决定是否参加本项研究，也可以和您的家人或者朋友商量后再做出决定。在您做出参加研究的决定前，请尽可能向研究人员了解本项目的具体情况，直至您对本项研究完全理解。感谢您阅读以上材料。如果您决定参加本项研究，请尽快告知研究人员，我们会为您安排一切有关的具体事宜。

请您保留这份资料。

中国科学院上海生命科学研究院营养科学研究所

2006年3月

知情同意书·同意签字页

临床研究项目名称：亚麻子健康改善项目—植物木酚素对2型糖尿病患者代谢的影响及机制研究

研究者：中国科学院上海生命科学研究院营养科学研究所；上海华东医院

伦理审查批件号：**中科院上海生科院营养科学研究所伦理审查委员会E-2006-02**

同意声明

我已经阅读了上述有关本项目的介绍，而且有机会就此项目与研究人员讨论并提出问题。我提出的所有问题都得到了满意的答复。

我了解参加本研究可能产生的受益和风险。我知晓参加研究是自愿的，我确认已有充足时间对此进行考虑，而且愿意做到以下几点：

- 我愿意按研究项目的要求按时按量服用两个阶段的胶囊。
- 我声明在参与此次试验时，本人没有严重心脑血管疾病、严重甲状腺和垂体疾病、严重胃肠疾病、神经精神系统疾病和传染病，肝肾功能正常，不使用胰岛素控制糖尿病。
- 我了解我可以随时退出研究。如果我中途退出研究，特别是由于身体健康的原因退出时，我会将病情变化告诉研究人员，完成相应的体格检查和实验室生化检查。
- 如果因病情变化我需要采取任何其他的药物治疗，我会在事先征求研究人员的意见，或在事后如实告诉研究人员。

我将获得一份经过签名并注明日期的知情同意书副本。

最后，我决定同意参加本项研究，并尽量遵从研究人员的安排。

受试者签名：____________________ 日期：_________年______月______日

受试者联系电话：____

研究者声明

我确认已向受试者解释了本研究的详细情况，包括其权力以及可能的受益和风险，并给其一份签署过的知情同意书副本。

研究者签名：____________________ 日期：_________年______月______日

附录 3 体检现场组织标准操作规程

体检操作流程：

一、体检前通知与准备

1. 入户或集中调查完成《一般调查问卷》，指导受试者如何填写《3天膳食记录》，并于体检前一周内的三天（包括两个工作日和一个周末）完成记录。提前发放尿样保存瓶。
2. 通知受试者体检时间和注意事项以及需要带来的东西（如未服用完的胶囊的药瓶，病历卡，服用药物，尿样保存瓶等）；
3. 现场实验室准备、检验设备调试、质控样检测并上报结果；
4. 体格检查用物品、器材及场所准备；
5. 体检相关人员分组。

二、体检方案

1. 人员分工（共25人）

1.1 组织协调：工作人员1人

1.2 回收未服用完的胶囊瓶和发放新胶囊：工作人员1人；
回收和确认三天膳食记录表格：工作人员1名；

1.3 体检表、采血管袋发放：工作人员1人；

1.4 采血：工作人员6人（1人采血，2人负责血糖测定，3人负责分离血浆和血清）；

1.5 尿样收集：工作人员2人（1人发放和回收尿样保存瓶及加入防腐剂，1人负责分装）；

1.6 身高、体重、腰围、臀围测量：工作人员4名；血压测量：工作人员2名；

1.7 收集体检表和受试者手册，登记体检结果于受试者手册中：工作人员1名；

1.8 发放早餐，发放补偿费：工作人员1名；

1.9 受试者病例表（CRF）询问和调查问卷询问：工作人员6名左右；

2.0 体检现场中至少有一名内科医生。

2. 体检场所要求

2.1 血压测定需要独立房间，并保持房间安静，室温控制在20℃左右；

2.2 体重、腰臀围测量需要独立房间或有屏风隔离，室温控制在20℃左右,以方便调查对象脱去外衣。实行男女分开测定；

2.3 采血样需要独立房间，室温控制在20℃左右；

2.4 尿样采集厕所要求宽敞、干净，经常打扫和消毒。

三、工作流程

3.1 收集未服用完的胶囊；收集三天膳食记录表格。

3.2 收集尿样；

3.3 采集受试者静脉血；

3.4 测量身高、体重、腰围、臀围；

3.5 测定血压；

3.6 体格检查表上交，反馈体检结果于受试者手册上，发放早餐；

3.7 CRF或问卷调查；

3.8 发放新胶囊；发放营养费或补偿费。

四、体检表、胶囊、血样和尿样转运

填写转运清单，将体检表、胶囊、血样和尿样分别打包后转运至上海营养所。

五、体检及实验室结果反馈

体格检查结果在体检完成后，现场立即反馈在《受试者手册》上。如受试者没有携带《受试者手册》，也可在事后电话反馈给受试者，或者在受试者下次随访前往华东医院时反馈给受试者。

四次血样和尿样的相关生化指标分析将在整个临床干预结束之后在中科院营养所按照国际通用的方法进行测定，血糖、血脂、胰岛素、糖化血红蛋白等常规指标将在测定完成后及时反馈给每个受试者。

附录 4 随访现场组织标准操作规程

随访操作流程：

一、随访前通知与准备

1. 提前通知受试者随访时间、地点和注意事项以及需要带来的东西（如未服用完的胶囊的药瓶，病历卡，随访之间和正在服用的药物等）；
2. 随访现场体检用物品、器材及场所准备；
3. 体检相关人员分组。

二、随访方案

1. 人员分工（共5人）

1.1 组织协调：工作人员1人

1.2 回收未服用完的胶囊瓶和发放新胶囊：工作人员1人；

1.3 血糖测定：工作人员1人；

1.4 体重测定和血压测量：工作人员1名；

1.5 受试者病例表（CRF）询问：工作人员1名；

2. 随访场所要求

2.1 血压测定需要独立房间，并保持房间安静，室温控制在20℃左右；

2.2 体重测定室温控制在20℃左右，以方便调查对象脱去外衣；

2.3 血糖测定室温控制在20℃左右。

三、工作流程

3.1 收集未服用完的胶囊；

3.2 测定血压；

3.3 血糖检测；

3.4 测量体重；

3.5 反馈体检结果至受试者手册中，受试者病例表（CRF）询问。

3.6 发放新胶囊（体检前的那次面对面随访需要发放3天膳食记录表格和尿样保存瓶）。

附录五 体检表

姓名：	编号：
性别：	体检日期:2006 年___月___日
检查员:	质控员:

亚麻子健康改善计划

（体 检 表）

1. 空腹：☐是 ☐否

空腹时间：__________小时

2. 留尿：☐完成 ☐未完成

3. 血糖：☐☐.☐毫摩尔/升

4. 身高：☐☐☐.☐厘米

（精确到 0.1 厘米）

5. 体重：☐☐☐.☐公斤

（精确到 0.1 公斤）

6. 腰围:☐☐☐.☐厘米

（精确到 0.1 厘米）

7. 臀围:☐☐☐.☐厘米

（精确到 0.1 厘米）

8. 血压和脉搏：

第一次测量：

收缩压☐☐☐毫米汞柱

舒张压☐☐☐毫米汞柱

脉 搏☐☐☐/分钟

第二次测量：

收缩压☐☐☐毫米汞柱

舒张压☐☐☐毫米汞柱

脉 搏☐☐☐/分钟

第三次测量：

收缩压☐☐☐毫米汞柱

舒张压☐☐☐毫米汞柱

脉 搏☐☐☐/分钟

9. 一般问卷: ☐完成 ☐未完成

10. 三天膳食:☐完成 ☐未完成

11. 药物登记:☐完成 ☐未完成

12. 膳食频率:☐完成 ☐未完成
